# Network Pharmacology-Based Study on the Active Ingredients and Mechanism of Pan Ji Sheng Traditional Chinese Medicine Formula in the Treatment of Inflammation

**DOI:** 10.1155/2022/5340933

**Published:** 2022-09-28

**Authors:** Shiji Wu, Hongliang Jiang, Zongwen Chen, Weining Lu, Qin Chen

**Affiliations:** Gaozhou Hospital of Traditional Chinese Medicine, No. 32 Maoming Avenue, Gaozhou 525200, Guangdong, China

## Abstract

**Background:**

Pan Ji Sheng Formula is a Chinese medicine formula that enables heat-free detoxification as well as anti-inflammatory and immune-boosting properties. This formula contains eight herbs. Its underlying mechanism is unknown. The bioactive ingredients were screened in our work, and the mechanism of this formula was investigated.

**Methods:**

Using traditional Chinese medicine systems pharmacology database and analysis platform (TCMSP), ingredients in Pan Ji Sheng Chinese medicine formula were screened, and we selected the main bioactive ingredients for web-based research. The targets of bioactive ingredients are primarily obtained from the SwissTargetPrediction and TCMSP databases, and the text mining method is used. STRING and Cytoscape were then used to examine the protein-protein interaction (PPI) networks. To explore the biological function and related pathways, functional annotation and pathway analysis were performed.

**Results:**

This research discovered 96 bioactive ingredients. Then, 215 potential targets of bioactive ingredients were screened. Through the analysis of the PPI network, we discovered 25 key target genes, which can be described as hub target genes regulated by bioactive ingredients. Bioactive ingredients primarily regulate CASP3, AKT1, JUN, and other proteins. The formula works synergistically to enhance immune response and antiinfection by regulating immune-related pathways, TNF signaling pathways, and apoptosis.

**Conclusions:**

A variety of bioactive ingredients in the formula could play roles in regulating CASP3, AKT1, and other genes in immune, infection, apoptosis, and tumor-related signaling pathways. Our data point the way forward for future studies on the mechanism of action of this formula.

## 1. Introduction

The climate in China's Lingnan region is standard subtropical. Summers are hot, rainy, as well as wet [1]. Furthermore, Cantonese people prefer to eat fried, dry, and hot foods. It is easy to make people “heat” and “dampness” due to the hot and humid climate, poor diet, and insufficient sleep [2, 3]. The symptoms of “heat” contain fever, thirst, sweating, fatigue, yellow urine, and yellow tongue. The common symptoms of “dampness” contain head pain, chest tightness, sluggishness, and sore or swollen joints. “Heat” and “dampness” are considered to be the cause of many inflammatory disease, cancer, and metabolic disorders [2].

Inflammation is a pathological defense response and it is also the most important protective response [4]. In modern western medicine, clinical experimental data show that the current conventional treatment for inflammation is anti-inflammatory drugs and antibiotic drugs [5, 6]. Nonsteroidal anti-inflammatory drugs (NSAIDs) are extensively used to reduce inflammation [7]. NSAIDs, such as aspirin and ibuprofen, are effective by inhibiting cyclooxygenase (COX) activity, thereby suppressing inflammatory responses [8]. Although it is effective, some anti-inflammatory drugs can lead to some side effects, such as gastrointestinal damage, gastrointestinal bleeding, and cardiovascular risk [9, 10]. The long-term use of antibiotic drugs can also lead to drug-resistance and seriously affect the treatment effect [11]. Traditional Chinese medicine (TCM) has the advantages of long efficacy and safety, so it is necessary to excavate the TCM compound formulas for treating inflammation.

The ancestors attempted to collect herbs for clearing heat and detoxification, and boiling water for drinking to eliminate the “heat” in order to get rid of dampness and heat and adapt to the environment. Since this type of herbal medicine was safe to drink, it gradually spread among the people [12, 13]. People gradually dig up various therapeutic properties of traditional Chinese medicine substances under the research of ancient and modern science, and make formulas with heat-clearing and detoxification features with honeysuckle, Scutellaria baicalensis, chrysanthemum, isatis root, and other traditional Chinese medicines, so as to enhance immune response and alleviate problems such as getting angry and heavy moisture caused by improper diet and lack of sleep [14, 15]. TCM (traditional Chinese medicine) is a type of traditional medicine. TCM is still a vital resource with such a long history. TCM can still influence the advancement of modern medicine [16, 17]. The Pan Ji Sheng formula, which contains eight different herbs, is the subject of this research: *Microctis Folium* (the leaves of *Microcos paniculata)*, *Polygonum chinense* (creeping smartweed), *Ecliptae Herba* (false daisy), *Perilla Frutescens* (the leaves of Beefsteak Plant), *Isatidis Radix* (the dried roots of the plant *Isatis indigotica Fort* or *Isatis tinctoria L.*), *Chrysanthemi Flos* (the flower of *Chrysanthemum indicum Linne* or *Chrysanthemum morifolium Ramatuelle*), *Glycyrrhiza uralensis* (Chinese liquorice, the root of *Glycyrrhiza uralensis*), and *Chimonanthus salicifolius* (wintersweet). All of these herbs are commonly used to treat diseases by clinicians. According to published research, these Chinese herbal medicines can prevent and treat diseases by utilizing a wide range of chemical components and multiple targets [18–21]. For example, isatis root lectin can directly kill influenza viruses by blocking the expression of nuclear proteins of new influenza viruses [22]; at the same time, nucleoside components such as uridine, guanosine, and adenosine can interfere with the synthesis of viral nucleic acid and perform critical roles for influenza virus defense [23], and polysaccharides have immunomodulatory effects and play indirect roles for influenza virus defense [24].

There is, however, no systematic research report on the specific formula and network mechanism of the formula's effects of clearing heat, detoxifying, anti-inflammatory, and enhancing immune response. Now, researchers have realized the “one key, one lock” model is insufficient for deciphering drug effects, particularly in complex diseases [25]. Network pharmacology is a new technology that uses the receptor theory and biological network technology to elucidate drug action mechanisms [26]. Its research mode of “multicomponent network target action” opens up a new research field and its compound prescriptions with multicomponent and multitarget synergy [27]. Furthermore, the rapid development of biomedical data, such as the TCMSP (traditional Chinese medicine system pharmacology database and analysis platform), has facilitated such research [28]. As a result, web-based pharmacological analysis can provide us with a thorough understanding of the significance of each component, target, and pathway. Based on the research concept of traditional Chinese medicine's multicomponent and multitarget effect, this study explains the biological mechanism of clearing heat, detoxifying, anti-inflammatory, and enhancing immune response by using the network pharmacology technology and analyzing the target characteristics, biological function, and pathway of the Pan Ji Sheng formula. Our research provides a scientific basis for experimental research and product development.

## 2. Methods

### 2.1. Screening of Bioactive Ingredients

Through TCMSP, we search the relevant information about the bioactive ingredients in eight herbals in Pan Ji Sheng formula and screen the qualified compounds as the formula's active ingredients. The screening conditions are oral bioavailability (OB) ≥ 30%, number of hydrogen bond donors (Hdon) < 5, lipid water partition coefficient (Alogp) < 5, number of hydrogen bond receptors (HACC) < 10, intestinal epithelial permeability (Caco-2) > 0, drug class (DL) ≥ 0.18, and drug half-life (HL) ≥ 4. We obtained bioactive ingredients of six herbals (*Microctis Folium*, *Ecliptae Herba*, *Perilla Frutescens*, *Isatidis Radix*, *Chrysanthemi Flos*, and *Glycyrrhiza uralensis*) from the TCMSP database. There is no information about *Polygonum chinense* and *Chimonanthus salicifolia* in the TCMSP database, so we search the literature for bioactive ingredients of these two herbals, then test OB ≥ 30% and DL ≥ 0.18 in TCMSP to determine the active ingredients.

### 2.2. Target Prediction of Bioactive Ingredients

The formula's bioactive ingredients were imported to TCMSP to obtain information on ingredient-target interaction. Second, we use the Swiss Target Prediction online analysis tool to predict the active ingredient's targets, screen potential targets, extract the names of the target genes, and build the chemical ingredient-target interaction network. The specific method is to convert all ingredients into standard smiles format and import the smiles format file into the Swiss Target Prediction online analysis platform [29], set the species to “*Homo sapiens*,” and set Probability ≥0.7, and export the target data in the CSV format.

The target genes were then imported to the UniProt database to confirm their gene names. Through computer research, this study obtained the list of target genes for the traditional Chinese medicine Pan Ji Sheng formula.

### 2.3. Construction of the Protein-Protein Interaction (PPI) Network

We import target genes into STRING [30] and set the species to “*Homo sapiens* (human)” and use a confidence level of 0.9 to build the target interaction network (PPI). We hide the discrete points in the network, then export the results to a TSV file and import it to Cytoscape 3.9.1 [31]. Cytoscape was then used to construct the target's PPI network.

Then, in Cytoscape, the MCODE and Cytohubba plug-ins were used to extract the functional modules and top 25 hub genes of the PPI network, respectively.

### 2.4. Gene Ontology (GO) Functional Annotation and KEGG Pathway Analysis

All screened target genes were entered into the Metascape platform for enrichment analysis [32].

The hub targets were imported into the David database to clarify their function and role in signal transduction. GO biological process enrichment analysis and KEGG signal pathway analysis are carried out. The enrichment analysis results are enhanced with the R program package and displayed in the form of a bubble diagram.

### 2.5. Construction of the Bioactive Ingredients-Hub Target Network

Cytoscape 3.9.1 software was used to build the bioactive ingredients-hub target network. In this network, nodes represent bioactive ingredients and hub targets.

#### 2.5.1. Hub Target-GO BP/Pathway/Disease Network

Use Cytoscape 3.9.1 to build the network model. Nodes represent hub targets, pathways, and diseases, and edges represent interactions between these nodes.

## 3. Results

### 3.1. Screening of Bioactive Ingredients of the Pan Ji Sheng Formula

The bioactive ingredients of eight Chinese herbal medicines from the Pan Ji Sheng formula were screened from the TCMSP platform in this study. Because there is no relevant information on the TCMSP platform for *Polygonum chinense* and *Chimonanthus salicifolia*, we obtained the active components of these two herbals through literature retrieval and then tested whether they meet the standards of oral bioavailability (OB) ≥ 30 percent and drug class (DL) ≥ 0.18 in TCMSP. We obtained the active components of the other six herbals from TCMSP. In total, this study screened 96 active ingredients from eight herbals in the Pan Ji Sheng formula (Table 1).

### 3.2. Screening of Target Genes

Target genes of bioactive components were obtained using the TCMSP platform and Swiss target prediction screening. After removing the repeated target genes, we obtained a total of 214 target genes in this study (Table 2). For details of target genes, see Table S1.

### 3.3. Enrichment Analysis of All Target Genes

**Figure 1:**
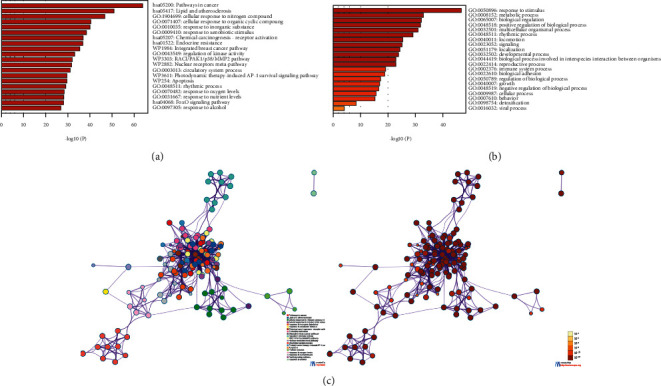
Enrichment analysis for bioactive ingredient targets by Metascape website. (a, b) Top 20 clusters with their representative enriched terms. (c) :Enrichment heatmap of the selected GO parents.

Using the Metascape website, this study firstly discovered relevant significantly enriched GO/KEGG terms for all target genes.  Figure 1 depicts the findings of the analysis. Many target genes are enriched in cancer and lipid metabolism-related pathways (Figures 1(a) and 1(b)). A subset of enriched terms was chosen and rendered as a network plot to further capture the relationships between the terms (Figure 1(c)).

**Figure 2:**
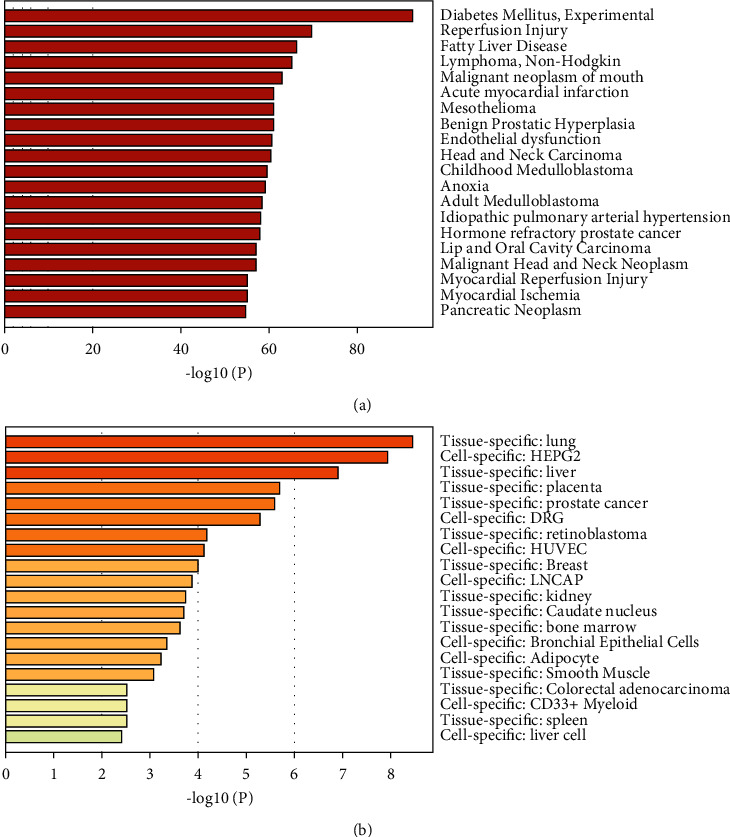
Related diseases and expression patterns of all target genes. (a) The summary of enrichment analysis in Disgenet. (b) The summary of enrichment analysis in PaGenBase.

We also analyzed related diseases and expression patterns of all target genes through Metascape, as shown in Figure 2. Diabetes, reperfusion injury, and fatty liver disease are the three most common diseases associated with target genes. The tissues that expressed the target genes were the lung and liver. According to preliminary findings, the target gene may be linked to lung and liver diseases.

#### 3.3.1. PPI Network for All Targets

We upload the names of all target genes to STRING. According to network statistics, the number of nodes is 214, the number of edges is 3057, and the average node degree is 28.6. The expected number of edges is 1173, and the local clustering coefficient is 0.583. We discovered that the network had far more interactions than expected. This suggests that the target proteins as a group are at least partially biologically connected.

**Figure 3:**
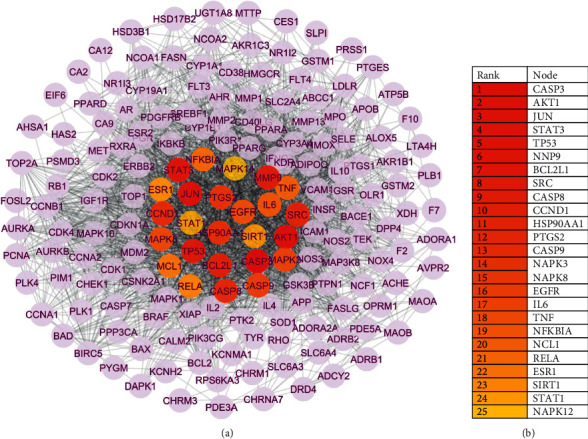
PPI network of all target genes. (a) PPI network, colored and in the middle are 25 hub genes. (b) Top 25 genes in the network ranked by the MCC method in “Cytohubba”.

Using Cytoscape 3.9.1, we constructed a PPI network (Figure 3(a)). Then, using the Cytoscape plug-in “cytohubba,” we analyzed hub targets and chose the top 25 target genes as hub genes (Figure 3(b)). CASP3, AKT1, Jun, STAT3, TP53, MMP9, BCL2l1, SRC, and other proteins. The higher the rank, the more important these target genes are in disease treatment. Hub targets are painted red and located at the center of the network for further analysis and research.

**Figure 4:**
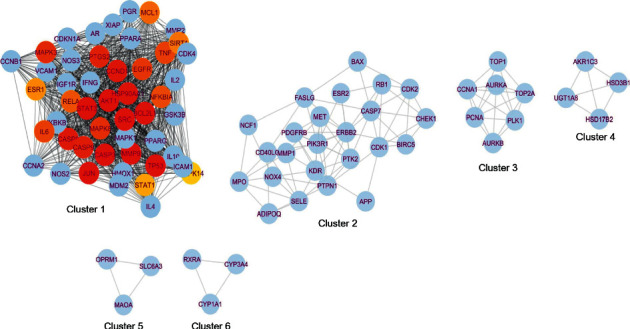
Clusters 1–6 in the PPI network. Among them, 25 hub genes are painted red and orange.

We also used the Cytoscape plug-in “MCODE” to examine the PPI network clusters and modules of all target genes (Figure 4). The PPI network is divided into six clusters, with 25 hub target genes located in Cluster 1, indicating that hub genes have biological function relevance and may play a synergistic role.

### 3.4. Herbal-Key Bioactive Ingredient-Hub Target Network

**Figure 5:**
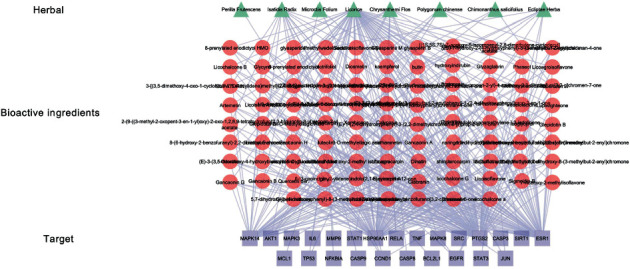
Herbal-key bioactive ingredient-hub target network.

After obtaining the hub target genes, we analyzed the active ingredients corresponding to these 25 hub genes, which are named as key bioactive ingredients. For more information, see Table S2. The network of herbal-key bioactive ingredient-hub targets was constructed using Cytoscape 3.9.1 (Figure 5). In addition to Perilla frutescens, the other seven Chinese herbal medicines have three or more corresponding key bioactive ingredients. Some hub genes are affected by multiple bioactive ingredients at the same time. The primary targets of the active ingredients are MAPK14, HSP90AA1, PTGS2, and ESR1. These genes may be the primary targets of the formula.

### 3.5. GO Functional Annotation and KEGG Pathway Analysis

To investigate the biological processes engaged in hub targets, GO enrichment analysis and KEGG enrichment analysis on 25 hub genes were analyzed in the David website. The mechanism of action of the formula can be researched, based on the biological process regulated by the hub target.

**Figure 6:**
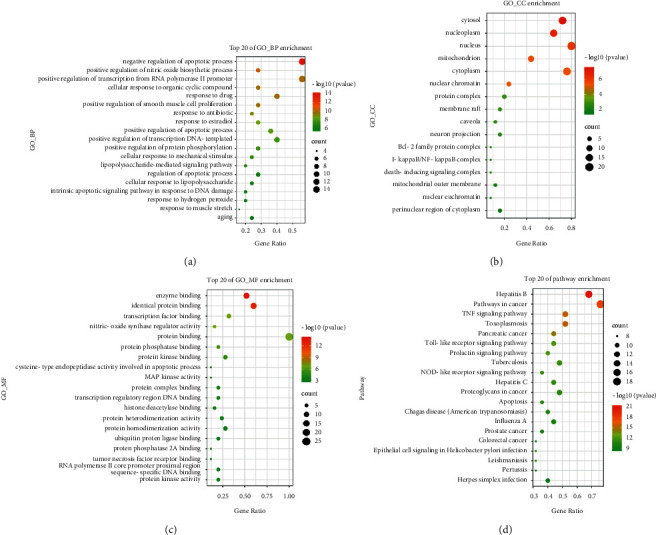
GO and KEGG enrichment analysis of hub genes.

Beautify the enrichment analysis results with *R* (Figure 6). In total, 226 GO biological process enrichment results were obtained. Negative regulation of the apoptotic process, positive regulation of the nitric oxide biosynthetic process, and positive regulation of transcription from the RNA polymerase II promoter are the top three enrichment biological processes. As shown in Figure 6(a), the top 20 GO biological processes are represented in the form of a bubble diagram, where the size of the circle represents the enrichment of relevant targets in the pathway, and the darker the color of the circle represents the degree of enrichment of targets, indicating that the formula could have physiological effects by regulating these biological processes.

For KEGG pathway enrichment analysis, 25 hub targets were mapped into the David database. The species was defined as “human,” and a total of 94 pathways were obtained. As shown in Figure 6(d), the top 20 pathways with high significance of KEGG enrichment results are closely related to the mechanism of the Pan Ji Sheng formula. The top five pathways include hepatitis B, pathways in cancer, TNF signaling pathway, toxoplasmosis, and toll-like receptor signaling pathway. The majority of these pathways are linked to the genes TP53, JUN, AKT1, MAPK14, HSP90AA1, and PTGS2.

**Figure 7:**
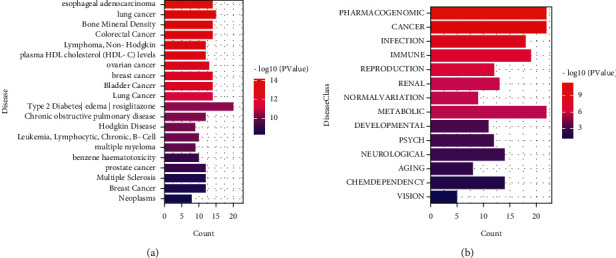
Disease and disease class enrichment analysis of hub genes.

We also performed disease enrichment analysis to investigate diseases associated with hub targets.  Figure 7 shows the classification of diseases enriched in hub targets. The three major categories are cancer, infection, and immune system. Our findings indicate that the formula studied in this study may primarily target these diseases.

#### 3.5.1. Hub Target-GO BP/Pathway/Disease Class Network

**Figure 8:**
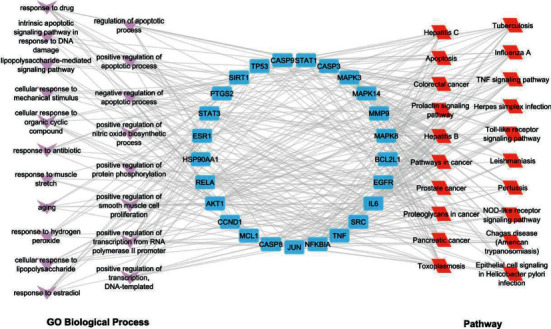
Hub target-GO BP/pathway network.

In order to demonstrate the biological process of the hub target and the relationship between the hub target and the pathway more clearly, the hub target-GO BP/pathway/disease class network was built with Cytoscape 3.9.1 software (Figure 8).

The hub target is represented by the circle in the center of Figure 8. The left and right sides of Figure 8 show the top 20 enriched biological processes and pathways, respectively. We can clearly understand the relationship between the targets and biological processes or pathways. MAPK14, hSP90AA1, and PTGS2 genes are associated with apoptotic biological processes, TNF signaling pathways, toll-like receptor signaling pathways, and cancer pathways. The formula could play a significant role by regulating these pathways.

**Figure 9:**
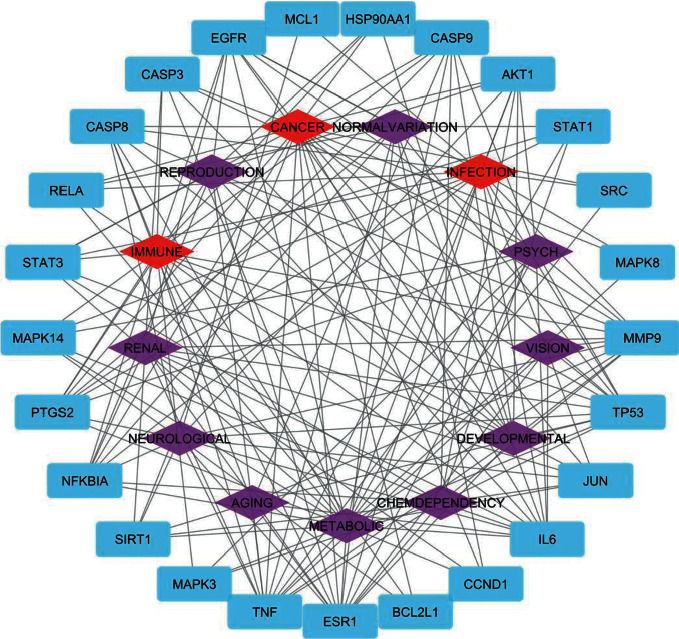
Hub target-disease class network.

In order to demonstrate the link between the hub targets and diseases more clearly, Cytoscape 3.9.1 software was used to create a network of hub targets and diseases (Figure 9). The genes MAPK14, HSP90AA1, PTGS2, and ESR1 have been linked to cancer, infection, and immune disease.

## 4. Discussion

Traditional Chinese medicine formulas are typically difficult to decipher due to the action mode of traditional Chinese medicine formulas [33]. Using network pharmacology, this study explains the action mechanism of the Pan Ji Sheng Chinese medicine formula. According to the findings of this study, CASP3, AKT1, JUN, and other genes are the hub targets of the formula to enhance immune response and anti-inflammatory.

According to the active ingredient-target network, HSP90AA1, PTGS2, ESR1, and MAPK14 are the four key genes regulated by the active ingredient of the Pan Ji Sheng formula. HSP90AA1 is an inflammation-related protein that can be significantly upregulated with some inflammation-related genes in the inflammatory response [34, 35]; PTGS2 is involved in inflammation, immunity, and other processes [36, 37]; ESR1 is also involved in inflammation and immunity and is one of the key targets for the treatment of pneumonia [38, 39]; and MAPK14 is related to autophagy and plays an important role in immune response [40].

As shown in the results, 19 of the 25 hub targets were discovered to be involved in the pathways in cancer, with the pathways in cancer being the most significant pathway. This could be due to the fact that respiratory inflammation and lung disease are risk factors for cancer [41, 42]. Other top KEGG enrichment pathways include hepatitis B, the TNF signaling pathway, toxoplasmosis, and the toll-like receptor signaling pathway. A key target gene is tumor necrosis factor (TNF), a cytokine secreted by macrophages and adipocytes. It can cause IR by suppressing the activity of the PI3K/Akt signaling pathway. TNF has been shown to activate MAPK and NF-B signaling pathways, which regulate inflammatory response, oxidative stress, and apoptosis [43, 44].

The network pharmacological analysis reveals that the Pan Ji Sheng formula could regulate HSP90AA1, PTGS2, ESR1, MAPK14, and other genes, modulating pathways such as cancer pathways, TNF signaling pathways, and toll-like receptor signaling pathways to regulate inflammatory response and immune processes.

This study investigated the anti-inflammatory and immune mechanisms of Pan Ji Sheng formula. However, in vivo and in vitro experiments are needed to provide more information on the mechanism of action of the formula.

## 5. Conclusions

The active components of the Pan Ji Sheng formula could regulate certain proteins, including HSP90AA1, PTGS2, ESR1, and MAPK14. The Chinese herbs in the Pan Ji Sheng formula have a synergistic therapeutic effect, primarily by acting on inflammation and immune-related signal pathways. Pan Ji Sheng formula plays the functions through multicomponents, multitargets (HSP90AA1, PTGS2, ESR1, MAPK14, and other hub targets), and multipathways (inflammation and immune-related signal pathways). These findings could serve as guidelines for future research into this formula. Based on the present study, functional experiments can be performed on animal models or human cells to validate the pharmacological mechanisms of the Pan Ji Sheng formula in the future. This research has theoretical significance for the TCM pharmacology and has application value for the development and utilization of TCMs.

## Figures and Tables

**Table 1:** Herbal and bioactive ingredients of Pan Ji Sheng formula.

Herbals	Molecule names
*Microctis folium*	Isorhamnetin
	Kaempferol
	4′,5-Dihydroxyflavone
	Kaempferol
	Quercetin

*Polygonum chinense*	3-O-Methylellagic acid
	Kaempferol-7-O-glucoside
	3,3′-Di-O-methylellagic acid
	Protocatechuic acid
	Isorhamnetin
	Luteolin
	Acacetin

*Ecliptae herba*	Butin
	1,3,8,9-Tetrahydroxybenzofurano [3,2-c] chromen-6-one
	3′-O-Methylorobol
	Pratensein
	Demethylwedelolactone
	Wedelolactone
	Luteolin

*Perilla frutescens*	Luteolin
	Acacetin
	Eupatorin
	Dinatin
	Quindoline
	Hydroxyindirubin
	Indigo
	(2Z)-2-(2-Oxoindolin-3-ylidene) indolin-3-one

*Isatidis radix*	2-(9-((3-Methyl-2-oxopent-3-en-1-yl) oxy)-2-oxo-1,2,8,9-tetrahydrofuro [2,3-h] quinolin-8-yl) propan-2-yl acetate
	DFV
	(E)-2-[(3-Indole) cyanomethylene-]-3-indolinone
	neohesperidin_qt
	Sinensetin
	6-(3-Oxoindolin-2-ylidene) indolo[2,1-b]quinazolin-12-one
	(E)-3-(3,5-Dimethoxy-4-hydroxy-benzylidene)-2-indolinone
	(E)-3-(3,5-Dimethoxy-4-hydroxyb-enzylidene)-2-indolinone
	3-[(3,5-Dimethoxy-4-oxo-1-cyclohexa-2,5-dienylidene)methyl]-2,4-dihydro-1H-pyrrolo[2,1-b] quinazolin-9-one
	[(1S,5S,7S)-7-Acetoxy-5-isopropenyl-2,8-dimethylene-cyclodecyl] acetate
	Acacetin
	Chryseriol
	Isorhamnetin

*Chrysanthemi flos*	Kaempferol
	5,7-Dihydroxy-2-(3-hydroxy-4-methoxyphenyl) chroman-4-one
	Luteolin
	Eupatorin
	Diosmetin
	Naringenin
	Artemetin
	Jaranol
	Isorhamnetin
	Formononetin

*Licorice*	Calycosin
	Kaempferol
	Licochalcone a
	Inermine
	DFV
	Glycyrol
	Medicarpin
	Lupiwighteone
	7-Methoxy-2-methyl isoflavone
	Naringenin
	Glyasperin B
	Glyasperin F
	Isotrifoliol
	(E)-1-(2,4-Dihydroxyphenyl)-3-(2,2-dimethylchromen-6-yl) prop-2-en-1-one
	(2S)-6-(2,4-Dihydroxyphenyl)-2-(2-hydroxypropan-2-yl)-4-methoxy-2,3-dihydrofuro [3,2-g] chromen-7-one
	Semilicoisoflavone B
	Glepidotin A
	Glepidotin B
	Glypallichalcone
	8-(6-Hydroxy-2-benzofuranyl)-2,2-dimethyl-5-chromenol
	Licochalcone B
	Licochalcone G
	Licoricone
	Gancaonin A
	Gancaonin B
	3-(3,4-Dihydroxyphenyl)-5,7-dihydroxy-8-(3-methylbut-2-enyl) chromone
	5,7-Dihydroxy-3-(4-methoxyphenyl)-8-(3-methylbut-2-enyl) chromone
	2-(3,4-Dihydroxyphenyl)-5,7-dihydroxy-6-(3-methylbut-2-enyl) chromone
	Licocoumarone
	Licoisoflavone
	Licoisoflavone B
	Licoisoflavanone
	Shinpterocarpin
	(E)-3-[3,4-Dihydroxy-5-(3-methylbut-2-enyl)phenyl]-1-(2,4-dihydroxyphenyl) prop-2-en-1-one
	Glyzaglabrin
	Glabranin
	Glabrone
	1,3-Dihydroxy-9-methoxy-6-benzofurano[3,2-c] chromenone
	1,3-Dihydroxy-8,9-dimethoxy-6-benzofurano[3,2-c] chromenone
	Eurycarpin A
	Sigmoidin-B
	(2R)-7-Hydroxy-2-(4-hydroxyphenyl) chroman-4-one
	(2S)-7-Hydroxy-2-(4-hydroxyphenyl)-8-(3-methylbut-2-enyl) chroman-4-one
	Isoglycyrol
	Isolicoflavonol
	HMO
	1-Methoxyphaseollidin
	Quercetin der.
	6-Prenylated eriodictyol
	7-Acetoxy-2-methylisoflavone
	8-Prenylated eriodictyol
	Gancaonin G
	Gancaonin H
	Licoagrocarpin
	Glyasperins M
	Licoagroisoflavone
	Odoratin
	Phaseol
	Xambioona

*Chimonanthus salicifolius*	Luteolin-5-O-glucoside
	Quercetin
	Kaempferol

**Table 2:** Potential target genes of bioactive ingredients of Pan Ji Sheng formula.

No.	Target gene names	String Id
1	NOS2	9606.ENSP00000327251
2	PTGS1	9606.ENSP00000354612
3	ESR1	9606.ENSP00000405330
4	AR	9606.ENSP00000363822
5	PPARG	9606.ENSP00000287820
6	PTGS2	9606.ENSP00000356438
7	PTPN1	9606.ENSP00000360683
8	ESR2	9606.ENSP00000343925
9	DPP4	9606.ENSP00000353731
10	MAPK14	9606.ENSP00000229795
11	GSK3B	9606.ENSP00000324806
12	HSP90AA1	9606.ENSP00000335153
13	CDK2	9606.ENSP00000266970
14	PIK3CG	9606.ENSP00000352121
15	PKIA	9606.ENSP00000379696
16	PRSS1	9606.ENSP00000308720
17	PIM1	9606.ENSP00000362608
18	CCNA2	9606.ENSP00000274026
19	NCOA2	9606.ENSP00000399968
20	CALM2	9606.ENSP00000272298
21	PYGM	9606.ENSP00000164139
22	PPARD	9606.ENSP00000310928
23	CHEK1	9606.ENSP00000388648
24	AKR1B1	9606.ENSP00000285930
25	NCOA1	9606.ENSP00000385216
26	F7	9606.ENSP00000364731
27	F2	9606.ENSP00000308541
28	NOS3	9606.ENSP00000297494
29	ACHE	9606.ENSP00000303211
30	GABRA1	9606.ENSP00000393097
31	MAOB	9606.ENSP00000367309
32	GRIA2	9606.ENSP00000296526
33	RELA	9606.ENSP00000384273
34	XDH	9606.ENSP00000368727
35	NCF1	9606.ENSP00000289473
36	OLR1	9606.ENSP00000309124
37	PGR	9606.ENSP00000325120
38	CHRM1	9606.ENSP00000306490
39	GABRA2	9606.ENSP00000421828
40	SLC6A2	9606.ENSP00000219833
41	CHRM2	9606.ENSP00000399745
42	ADRA1B	9606.ENSP00000306662
43	TOP2A	9606.ENSP00000411532
44	IKBKB	9606.ENSP00000430684
45	AKT1	9606.ENSP00000451828
46	BCL2	9606.ENSP00000381185
47	BAX	9606.ENSP00000293288
48	CD40LG	9606.ENSP00000359663
49	JUN	9606.ENSP00000360266
50	AHSA1	9606.ENSP00000216479
51	CASP3	9606.ENSP00000311032
52	MAPK8	9606.ENSP00000378974
53	MMP1	9606.ENSP00000322788
54	STAT1	9606.ENSP00000354394
55	CDK1	9606.ENSP00000378699
56	HMOX1	9606.ENSP00000216117
57	CYP3A4	9606.ENSP00000337915
58	CYP1A1	9606.ENSP00000369050
59	ICAM1	9606.ENSP00000264832
60	SELE	9606.ENSP00000331736
61	VCAM1	9606.ENSP00000294728
62	NR1I2	9606.ENSP00000336528
63	CYP1B1	9606.ENSP00000478561
64	ALOX5	9606.ENSP00000363512
65	HAS2	9606.ENSP00000306991
66	AHR	9606.ENSP00000242057
67	PSMD3	9606.ENSP00000264639
68	SLC2A4	9606.ENSP00000320935
69	NR1I3	9606.ENSP00000356959
70	INSR	9606.ENSP00000303830
71	DIO1	9606.ENSP00000354643
72	GSTM1	9606.ENSP00000311469
73	GSTM2	9606.ENSP00000241337
74	AKR1C3	9606.ENSP00000369927
75	SLPI	9606.ENSP00000342082
76	NOX4	9606.ENSP00000263317
77	AVPR2	9606.ENSP00000351805
78	MAOA	9606.ENSP00000340684
79	IGF1R	9606.ENSP00000268035
80	FLT3	9606.ENSP00000241453
81	CYP19A1	9606.ENSP00000379683
82	EGFR	9606.ENSP00000275493
83	CA2	9606.ENSP00000285379
84	AURKB	9606.ENSP00000313950
85	DRD4	9606.ENSP00000176183
86	ADORA1	9606.ENSP00000356205
87	CA7	9606.ENSP00000345659
88	GLO1	9606.ENSP00000362463
89	MPO	9606.ENSP00000225275
90	PIK3R1	9606.ENSP00000428056
91	ADORA2A	9606.ENSP00000336630
92	DAPK1	9606.ENSP00000386135
93	PYGL	9606.ENSP00000216392
94	CA1	9606.ENSP00000430656
95	SRC	9606.ENSP00000362680
96	PTK2	9606.ENSP00000341189
97	HSD17B2	9606.ENSP00000199936
98	KDR	9606.ENSP00000263923
99	MMP13	9606.ENSP00000260302
100	CA12	9606.ENSP00000178638
101	CA13	9606.ENSP00000318912
102	CA9	9606.ENSP00000367608
103	GPR35	9606.ENSP00000411788
104	ERBB2	9606.ENSP00000269571
105	CCND1	9606.ENSP00000227507
106	CDK4	9606.ENSP00000257904
107	PDGFRB	9606.ENSP00000261799
108	FLT4	9606.ENSP00000261937
109	CCNA1	9606.ENSP00000255465
110	PLK1	9606.ENSP00000300093
111	CA6	9606.ENSP00000366654
112	CA14	9606.ENSP00000358107
113	CSNK2A1	9606.ENSP00000217244
114	MET	9606.ENSP00000317272
115	CA4	9606.ENSP00000300900
116	PLK4	9606.ENSP00000270861
117	TEK	9606.ENSP00000369375
118	TNF	9606.ENSP00000398698
119	IL2	9606.ENSP00000226730
120	RPS6KA3	9606.ENSP00000368884
121	CD38	9606.ENSP00000226279
122	PDE5A	9606.ENSP00000347046
123	NQO2	9606.ENSP00000369822
124	ADRA2C	9606.ENSP00000386069
125	ALDH2	9606.ENSP00000261733
126	NMUR2	9606.ENSP00000255262
127	ADRA2A	9606.ENSP00000280155
128	SLC29A1	9606.ENSP00000377424
129	AURKA	9606.ENSP00000216911
130	CA5A	9606.ENSP00000309649
131	BACE1	9606.ENSP00000318585
132	MAP3K8	9606.ENSP00000263056
133	BRAF	9606.ENSP00000288602
134	BCL2L1	9606.ENSP00000302564
135	CDKN1A	9606.ENSP00000384849
136	CASP9	9606.ENSP00000330237
137	MMP2	9606.ENSP00000219070
138	MMP9	9606.ENSP00000361405
139	MAPK1	9606.ENSP00000215832
140	IL10	9606.ENSP00000412237
141	RB1	9606.ENSP00000267163
142	CDK4	9606.ENSP00000257904
143	IL6	9606.ENSP00000385675
144	TP53	9606.ENSP00000269305
145	NFKBIA	9606.ENSP00000216797
146	TOP1	9606.ENSP00000354522
147	MDM2	9606.ENSP00000258149
148	APP	9606.ENSP00000284981
149	PCNA	9606.ENSP00000368458
150	CASP7	9606.ENSP00000358327
151	MCL1	9606.ENSP00000358022
152	BIRC5	9606.ENSP00000301633
153	CCNB1	9606.ENSP00000256442
154	TYR	9606.ENSP00000263321
155	IFNG	9606.ENSP00000229135
156	IL4	9606.ENSP00000231449
157	XIAP	9606.ENSP00000360242
158	PTGES	9606.ENSP00000342385
159	NUF2	9606.ENSP00000271452
160	ADCY2	9606.ENSP00000342952
161	ADRB2	9606.ENSP00000305372
162	PDE3A	9606.ENSP00000351957
163	CASP8	9606.ENSP00000351273
164	FASN	9606.ENSP00000304592
165	FASLG	9606.ENSP00000356694
166	RXRA	9606.ENSP00000419692
167	LACTBL1	9606.ENSP00000402297
168	SCN5A	9606.ENSP00000410257
169	F10	9606.ENSP00000364709
170	RHO	9606.ENSP00000296271
171	KCNH2	9606.ENSP00000262186
172	KCNMA1	9606.ENSP00000286628
173	SLC6A4	9606.ENSP00000261707
174	CHRNA7	9606.ENSP00000407546
175	PPP3CA	9606.ENSP00000378323
176	MAPK3	9606.ENSP00000263025
177	LDLR	9606.ENSP00000454071
178	BAD	9606.ENSP00000378040
179	SOD1	9606.ENSP00000270142
180	MTTP	9606.ENSP00000427679
181	APOB	9606.ENSP00000233242
182	PLB1	9606.ENSP00000330442
183	HMGCR	9606.ENSP00000287936
184	UGT1A8	9606.ENSP00000304845
185	PPARA	9606.ENSP00000385523
186	SREBF1	9606.ENSP00000348069
187	GSR	9606.ENSP00000221130
188	ABCC1	9606.ENSP00000382342
189	ADIPOQ	9606.ENSP00000389814
190	SOAT2	9606.ENSP00000301466
191	AKR1C1	9606.ENSP00000370254
192	GOT1	9606.ENSP00000359539
193	ABAT	9606.ENSP00000379845
194	CES1	9606.ENSP00000353720
195	SOAT1	9606.ENSP00000356591
196	ADRA1D	9606.ENSP00000368766
197	SLC6A3	9606.ENSP00000270349
198	SIRT1	9606.ENSP00000212015
199	ATP5B	9606.ENSP00000262030
200	MT-ND6	9606.ENSP00000354665
201	HSD3B2	9606.ENSP00000445122
202	HSD3B1	9606.ENSP00000358421
203	STAT3	9606.ENSP00000264657
204	EIF6	9606.ENSP00000363574
205	FOSL2	9606.ENSP00000264716
206	CHRM3	9606.ENSP00000255380
207	OPRM1	9606.ENSP00000394624
208	DRD1	9606.ENSP00000377353
209	CHRM5	9606.ENSP00000372750
210	CHRM4	9606.ENSP00000409378
211	HTR2A	9606.ENSP00000437737
212	MAPK10	9606.ENSP00000352157
213	OPRD1	9606.ENSP00000234961
214	ADRB1	9606.ENSP00000358301
215	LTA4H	9606.ENSP00000228740

## Data Availability

The data used to support the findings of this study are included within the supplementary information files.

## Supplementary Materials

Supplementary Materials Table S1: targets of Pan Ji Sheng Formula. Table S2: detailed information of herbal-key bioactive ingredients-top 25 hub targets.
