# Supplementary material for: Network Pharmacology-Based Study on the Active Ingredients and Mechanism of Pan Ji Sheng Traditional Chinese Medicine Formula in the Treatment of Inflammation
Source: Evid Based Complement Alternat Med. 2022 Sep 28;2022:5340933. doi: 10.1155/2022/5340933 (PMC9534616; doi:10.1155/2022/5340933)
Supplement: Supplementary Materials — Table S1: targets of Pan Ji Sheng Formula. Table S2: detailed information of herbal-key bioactive ingredients-top 25 hub targets. [file 5340933.f1.zip › Table S1.pdf]

Herbal Medicine	Bioactive Ingredients	Target Proteins
Microctis Folium	isorhamnetin	NOS2
Microctis Folium	isorhamnetin	PTGS1
Microctis Folium	isorhamnetin	ESR1
Microctis Folium	isorhamnetin	AR
Microctis Folium	isorhamnetin	PPARG
Microctis Folium	isorhamnetin	PTGS2
Microctis Folium	isorhamnetin	PTPN1
Microctis Folium	isorhamnetin	ESR2
Microctis Folium	isorhamnetin	DPP4
Microctis Folium	isorhamnetin	MAPK14
Microctis Folium	isorhamnetin	GSK3B
Microctis Folium	isorhamnetin	HSP90AA1
Microctis Folium	isorhamnetin	CDK2
Microctis Folium	isorhamnetin	PIK3CG
Microctis Folium	isorhamnetin	PKIA
Microctis Folium	isorhamnetin	PRSS1
Microctis Folium	isorhamnetin	PIM1
Microctis Folium	isorhamnetin	CCNA2
Microctis Folium	isorhamnetin	NCOA2
Microctis Folium	isorhamnetin	CALM2
Microctis Folium	isorhamnetin	PYGM
Microctis Folium	isorhamnetin	PPARD
Microctis Folium	isorhamnetin	CHEK1
Microctis Folium	isorhamnetin	AKR1B1
Microctis Folium	isorhamnetin	NCOA1
Microctis Folium	isorhamnetin	F7
Microctis Folium	isorhamnetin	F2
Microctis Folium	isorhamnetin	NOS3
Microctis Folium	isorhamnetin	ACHE
Microctis Folium	isorhamnetin	GABRA1
Microctis Folium	isorhamnetin	MAOB
Microctis Folium	isorhamnetin	GRIA2
Microctis Folium	isorhamnetin	RELA
Microctis Folium	isorhamnetin	XDH
Microctis Folium	isorhamnetin	NCF1
Microctis Folium	isorhamnetin	OLR1
Microctis Folium	kaempferol	NOS2
Microctis Folium	kaempferol	PTGS1
Microctis Folium	kaempferol	AR
Microctis Folium	kaempferol	PPARG
Microctis Folium	kaempferol	PTGS2
Microctis Folium	kaempferol	HSP90AA1
Microctis Folium	kaempferol	PIK3CG
Microctis Folium	kaempferol	PKIA
Microctis Folium	kaempferol	NCOA2
Microctis Folium	kaempferol	DPP4
Microctis Folium	kaempferol	PRSS1
Microctis Folium	kaempferol	PGR
Microctis Folium	kaempferol	F2
Microctis Folium	kaempferol	CHRM1
Microctis Folium	kaempferol	NOS3
Microctis Folium	kaempferol	GABRA2
Microctis Folium	kaempferol	ACHE
Microctis Folium	kaempferol	SLC6A2
Microctis Folium	kaempferol	CHRM2
Microctis Folium	kaempferol	ADRA1B
Microctis Folium	kaempferol	GABRA1

Microctis Folium	kaempferol	TOP2A
Microctis Folium	kaempferol	F7
Microctis Folium	kaempferol	CALM2
Microctis Folium	kaempferol	RELA
Microctis Folium	kaempferol	IKBKB
Microctis Folium	kaempferol	AKT1
Microctis Folium	kaempferol	BCL2
Microctis Folium	kaempferol	BAX
Microctis Folium	kaempferol	CD40LG
Microctis Folium	kaempferol	JUN
Microctis Folium	kaempferol	AHSA1
Microctis Folium	kaempferol	CASP3
Microctis Folium	kaempferol	MAPK8
Microctis Folium	kaempferol	XDH
Microctis Folium	kaempferol	MMP1
Microctis Folium	kaempferol	STAT1
Microctis Folium	kaempferol	CDK1
Microctis Folium	kaempferol	PPARG
Microctis Folium	kaempferol	HMOX1
Microctis Folium	kaempferol	CYP3A4
Microctis Folium	kaempferol	CYP1A1
Microctis Folium	kaempferol	ICAM1
Microctis Folium	kaempferol	SELE
Microctis Folium	kaempferol	VCAM1
Microctis Folium	kaempferol	NR1I2
Microctis Folium	kaempferol	CYP1B1
Microctis Folium	kaempferol	ALOX5
Microctis Folium	kaempferol	HAS2
Microctis Folium	kaempferol	AHR
Microctis Folium	kaempferol	PSMD3
Microctis Folium	kaempferol	SLC2A4
Microctis Folium	kaempferol	NR1I3
Microctis Folium	kaempferol	INSR
Microctis Folium	kaempferol	DIO1
Microctis Folium	kaempferol	GSTM1
Microctis Folium	kaempferol	GSTM2
Microctis Folium	kaempferol	AKR1C3
Microctis Folium	kaempferol	SLPI
Microctis Folium	4',5-Dihydroxyflavone	PTGS1
Microctis Folium	4',5-Dihydroxyflavone	AR
Microctis Folium	4',5-Dihydroxyflavone	PTGS2
Microctis Folium	4',5-Dihydroxyflavone	DPP4
Microctis Folium	4',5-Dihydroxyflavone	HSP90AA1
Microctis Folium	4',5-Dihydroxyflavone	PIK3CG
Microctis Folium	4',5-Dihydroxyflavone	MAOB
Microctis Folium	4',5-Dihydroxyflavone	PKIA
Polygonum chinense	kaempferol	NOS2
Polygonum chinense	kaempferol	PTGS1
Polygonum chinense	kaempferol	AR
Polygonum chinense	kaempferol	PPARG
Polygonum chinense	kaempferol	PTGS2
Polygonum chinense	kaempferol	HSP90AA1
Polygonum chinense	kaempferol	PIK3CG
Polygonum chinense	kaempferol	PKIA
Polygonum chinense	kaempferol	NCOA2
Polygonum chinense	kaempferol	DPP4
Polygonum chinense	kaempferol	PRSS1
Polygonum chinense	kaempferol	PGR

Polygonum chinense	kaempferol	F2
Polygonum chinense	kaempferol	CHRM1
Polygonum chinense	kaempferol	NOS3
Polygonum chinense	kaempferol	GABRA2
Polygonum chinense	kaempferol	ACHE
Polygonum chinense	kaempferol	SLC6A2
Polygonum chinense	kaempferol	CHRM2
Polygonum chinense	kaempferol	ADRA1B
Polygonum chinense	kaempferol	GABRA1
Polygonum chinense	kaempferol	TOP2A
Polygonum chinense	kaempferol	F7
Polygonum chinense	kaempferol	CALM2
Polygonum chinense	kaempferol	RELA
Polygonum chinense	kaempferol	IKBKB
Polygonum chinense	kaempferol	AKT1
Polygonum chinense	kaempferol	BCL2
Polygonum chinense	kaempferol	BAX
Polygonum chinense	kaempferol	CD40LG
Polygonum chinense	kaempferol	JUN
Polygonum chinense	kaempferol	AHSA1
Polygonum chinense	kaempferol	CASP3
Polygonum chinense	kaempferol	MAPK8
Polygonum chinense	kaempferol	XDH
Polygonum chinense	kaempferol	MMP1
Polygonum chinense	kaempferol	STAT1
Polygonum chinense	kaempferol	CDK1
Polygonum chinense	kaempferol	PPARG
Polygonum chinense	kaempferol	HMOX1
Polygonum chinense	kaempferol	CYP3A4
Polygonum chinense	kaempferol	CYP1A1
Polygonum chinense	kaempferol	ICAM1
Polygonum chinense	kaempferol	SELE
Polygonum chinense	kaempferol	VCAM1
Polygonum chinense	kaempferol	NR1I2
Polygonum chinense	kaempferol	CYP1B1
Polygonum chinense	kaempferol	ALOX5
Polygonum chinense	kaempferol	HAS2
Polygonum chinense	kaempferol	AHR
Polygonum chinense	kaempferol	PSMD3
Polygonum chinense	kaempferol	SLC2A4
Polygonum chinense	kaempferol	NR1I3
Polygonum chinense	kaempferol	INSR
Polygonum chinense	kaempferol	DIO1
Polygonum chinense	kaempferol	GSTM1
Polygonum chinense	kaempferol	GSTM2
Polygonum chinense	kaempferol	AKR1C3
Polygonum chinense	kaempferol	SLPI
Polygonum chinense	Quercetin	NOX4
Polygonum chinense	Quercetin	AVPR2
Polygonum chinense	Quercetin	AKR1B1
Polygonum chinense	Quercetin	XDH
Polygonum chinense	Quercetin	MAOA
Polygonum chinense	Quercetin	IGF1R
Polygonum chinense	Quercetin	FLT3
Polygonum chinense	Quercetin	CYP19A1
Polygonum chinense	Quercetin	EGFR
Polygonum chinense	Quercetin	F2
Polygonum chinense	Quercetin	CA2

Polygonum chinense	Quercetin	PIM1
Polygonum chinense	Quercetin	ALOX5
Polygonum chinense	Quercetin	AURKB
Polygonum chinense	Quercetin	DRD4
Polygonum chinense	Quercetin	ADORA1
Polygonum chinense	Quercetin	CA7
Polygonum chinense	Quercetin	GLO1
Polygonum chinense	Quercetin	MPO
Polygonum chinense	Quercetin	PIK3R1
Polygonum chinense	Quercetin	ADORA2A
Polygonum chinense	Quercetin	DAPK1
Polygonum chinense	Quercetin	PYGL
Polygonum chinense	Quercetin	CA1
Polygonum chinense	Quercetin	GSK3B
Polygonum chinense	Quercetin	SRC
Polygonum chinense	Quercetin	PTK2
Polygonum chinense	Quercetin	HSD17B2
Polygonum chinense	Quercetin	KDR
Polygonum chinense	Quercetin	MMP13
Polygonum chinense	3-O-methylellagic acid	CA7
Polygonum chinense	3-O-methylellagic acid	CA12
Polygonum chinense	3-O-methylellagic acid	CA13
Polygonum chinense	3-O-methylellagic acid	CA9
Polygonum chinense	3-O-methylellagic acid	GPR35
Polygonum chinense	3-O-methylellagic acid	ERBB2
Polygonum chinense	3-O-methylellagic acid	AKR1B1
Polygonum chinense	3-O-methylellagic acid	CCND1
Polygonum chinense	3-O-methylellagic acid	CDK4
Polygonum chinense	3-O-methylellagic acid	PDGFRB
Polygonum chinense	3-O-methylellagic acid	FLT4
Polygonum chinense	3-O-methylellagic acid	IGF1R
Polygonum chinense	3-O-methylellagic acid	INSR
Polygonum chinense	3-O-methylellagic acid	EGFR
Polygonum chinense	3-O-methylellagic acid	CA2
Polygonum chinense	3-O-methylellagic acid	CDK2
Polygonum chinense	3-O-methylellagic acid	CCNA1
Polygonum chinense	3-O-methylellagic acid	CCNA2
Polygonum chinense	3-O-methylellagic acid	AURKB
Polygonum chinense	3-O-methylellagic acid	CA1
Polygonum chinense	3-O-methylellagic acid	GSK3B
Polygonum chinense	3-O-methylellagic acid	SRC
Polygonum chinense	3-O-methylellagic acid	PTK2
Polygonum chinense	3-O-methylellagic acid	KDR
Polygonum chinense	3-O-methylellagic acid	PLK1
Polygonum chinense	3-O-methylellagic acid	CA6
Polygonum chinense	3-O-methylellagic acid	CA14
Polygonum chinense	3-O-methylellagic acid	CSNK2A1
Polygonum chinense	3-O-methylellagic acid	MET
Polygonum chinense	3-O-methylellagic acid	CA4
Polygonum chinense	3-O-methylellagic acid	PLK4
Polygonum chinense	3-O-methylellagic acid	TEK
Polygonum chinense	3-O-methylellagic acid	AKT1
Polygonum chinense	kaempferol-7-O-glucoside	TNF
Polygonum chinense	kaempferol-7-O-glucoside	AKR1B1
Polygonum chinense	kaempferol-7-O-glucoside	IL2
Polygonum chinense	kaempferol-7-O-glucoside	ADORA1
Polygonum chinense	kaempferol-7-O-glucoside	CA7
Polygonum chinense	kaempferol-7-O-glucoside	CA12

Polygonum chinense	kaempferol-7-O-glucoside	XDH
Polygonum chinense	kaempferol-7-O-glucoside	RPS6KA3
Polygonum chinense	kaempferol-7-O-glucoside	CA2
Polygonum chinense	kaempferol-7-O-glucoside	CA4
Polygonum chinense	kaempferol-7-O-glucoside	CD38
Polygonum chinense	kaempferol-7-O-glucoside	PDE5A
Polygonum chinense	kaempferol-7-O-glucoside	NQO2
Polygonum chinense	kaempferol-7-O-glucoside	NOX4
Polygonum chinense	kaempferol-7-O-glucoside	ADRA2C
Polygonum chinense	kaempferol-7-O-glucoside	ALDH2
Polygonum chinense	kaempferol-7-O-glucoside	ACHE
Polygonum chinense	kaempferol-7-O-glucoside	ALOX5
Polygonum chinense	kaempferol-7-O-glucoside	PTGS2
Polygonum chinense	kaempferol-7-O-glucoside	EGFR
Polygonum chinense	kaempferol-7-O-glucoside	NMUR2
Polygonum chinense	kaempferol-7-O-glucoside	ADRA2A
Polygonum chinense	kaempferol-7-O-glucoside	CA1
Polygonum chinense	kaempferol-7-O-glucoside	SLC29A1
Polygonum chinense	3,3'-Di-O-Methylellagic Acid	CA7
Polygonum chinense	3,3'-Di-O-Methylellagic Acid	CA12
Polygonum chinense	3,3'-Di-O-Methylellagic Acid	CA13
Polygonum chinense	3,3'-Di-O-Methylellagic Acid	CA9
Polygonum chinense	3,3'-Di-O-Methylellagic Acid	GPR35
Polygonum chinense	3,3'-Di-O-Methylellagic Acid	CCND1
Polygonum chinense	3,3'-Di-O-Methylellagic Acid	CDK4
Polygonum chinense	3,3'-Di-O-Methylellagic Acid	PDGFRB
Polygonum chinense	3,3'-Di-O-Methylellagic Acid	FLT4
Polygonum chinense	3,3'-Di-O-Methylellagic Acid	IGF1R
Polygonum chinense	3,3'-Di-O-Methylellagic Acid	INSR
Polygonum chinense	3,3'-Di-O-Methylellagic Acid	CA2
Polygonum chinense	3,3'-Di-O-Methylellagic Acid	CDK2
Polygonum chinense	3,3'-Di-O-Methylellagic Acid	CCNA1
Polygonum chinense	3,3'-Di-O-Methylellagic Acid	CCNA2
Polygonum chinense	3,3'-Di-O-Methylellagic Acid	AURKB
Polygonum chinense	3,3'-Di-O-Methylellagic Acid	CA1
Polygonum chinense	3,3'-Di-O-Methylellagic Acid	SRC
Polygonum chinense	3,3'-Di-O-Methylellagic Acid	PTK2
Polygonum chinense	3,3'-Di-O-Methylellagic Acid	KDR
Polygonum chinense	3,3'-Di-O-Methylellagic Acid	PLK1
Polygonum chinense	3,3'-Di-O-Methylellagic Acid	CA6
Polygonum chinense	3,3'-Di-O-Methylellagic Acid	CA14
Polygonum chinense	3,3'-Di-O-Methylellagic Acid	MET
Polygonum chinense	3,3'-Di-O-Methylellagic Acid	CA4
Polygonum chinense	3,3'-Di-O-Methylellagic Acid	PLK4
Polygonum chinense	3,3'-Di-O-Methylellagic Acid	TEK
Polygonum chinense	3,3'-Di-O-Methylellagic Acid	AKT1
Polygonum chinense	3,3'-Di-O-Methylellagic Acid	AURKA
Polygonum chinense	3,3'-Di-O-Methylellagic Acid	CA5A
Polygonum chinense	3,3'-Di-O-Methylellagic Acid	BACE1
Polygonum chinense	3,3'-Di-O-Methylellagic Acid	MAP3K8
Polygonum chinense	3,3'-Di-O-Methylellagic Acid	BRAF
Polygonum chinense	Protocatechuic Acid	CA2
Polygonum chinense	Protocatechuic Acid	CA7
Polygonum chinense	Protocatechuic Acid	CA1
Polygonum chinense	Protocatechuic Acid	CA6
Polygonum chinense	Protocatechuic Acid	CA12
Polygonum chinense	Protocatechuic Acid	CA14
Polygonum chinense	Protocatechuic Acid	CA9

Polygonum chinense	Protocatechuic Acid	CA4
Polygonum chinense	isorhamnetin	NOS2
Polygonum chinense	isorhamnetin	PTGS1
Polygonum chinense	isorhamnetin	ESR1
Polygonum chinense	isorhamnetin	AR
Polygonum chinense	isorhamnetin	PPARG
Polygonum chinense	isorhamnetin	PTGS2
Polygonum chinense	isorhamnetin	PTPN1
Polygonum chinense	isorhamnetin	ESR2
Polygonum chinense	isorhamnetin	DPP4
Polygonum chinense	isorhamnetin	MAPK14
Polygonum chinense	isorhamnetin	GSK3B
Polygonum chinense	isorhamnetin	HSP90AA1
Polygonum chinense	isorhamnetin	CDK2
Polygonum chinense	isorhamnetin	PIK3CG
Polygonum chinense	isorhamnetin	PKIA
Polygonum chinense	isorhamnetin	PRSS1
Polygonum chinense	isorhamnetin	PIM1
Polygonum chinense	isorhamnetin	CCNA2
Polygonum chinense	isorhamnetin	NCOA2
Polygonum chinense	isorhamnetin	CALM2
Polygonum chinense	isorhamnetin	PYGM
Polygonum chinense	isorhamnetin	PPARD
Polygonum chinense	isorhamnetin	CHEK1
Polygonum chinense	isorhamnetin	AKR1B1
Polygonum chinense	isorhamnetin	NCOA1
Polygonum chinense	isorhamnetin	F7
Polygonum chinense	isorhamnetin	F2
Polygonum chinense	isorhamnetin	NOS3
Polygonum chinense	isorhamnetin	ACHE
Polygonum chinense	isorhamnetin	GABRA1
Polygonum chinense	isorhamnetin	MAOB
Polygonum chinense	isorhamnetin	GRIA2
Polygonum chinense	isorhamnetin	RELA
Polygonum chinense	isorhamnetin	XDH
Polygonum chinense	isorhamnetin	NCF1
Polygonum chinense	isorhamnetin	OLR1
Polygonum chinense	luteolin	PTGS1
Polygonum chinense	luteolin	AR
Polygonum chinense	luteolin	PTGS2
Polygonum chinense	luteolin	HSP90AA1
Polygonum chinense	luteolin	PRSS1
Polygonum chinense	luteolin	NCOA2
Polygonum chinense	luteolin	PKIA
Polygonum chinense	luteolin	DPP4
Polygonum chinense	luteolin	PIK3CG
Polygonum chinense	luteolin	RELA
Polygonum chinense	luteolin	EGFR
Polygonum chinense	luteolin	AKT1
Polygonum chinense	luteolin	CCND1
Polygonum chinense	luteolin	BCL2L1
Polygonum chinense	luteolin	CDKN1A
Polygonum chinense	luteolin	CASP9
Polygonum chinense	luteolin	MMP2
Polygonum chinense	luteolin	MMP9
Polygonum chinense	luteolin	MAPK1
Polygonum chinense	luteolin	IL10
Polygonum chinense	luteolin	RB1

Polygonum chinense	luteolin	CDK4
Polygonum chinense	luteolin	CD40LG
Polygonum chinense	luteolin	JUN
Polygonum chinense	luteolin	IL6
Polygonum chinense	luteolin	CASP3
Polygonum chinense	luteolin	TP53
Polygonum chinense	luteolin	NFKBIA
Polygonum chinense	luteolin	XDH
Polygonum chinense	luteolin	TOP1
Polygonum chinense	luteolin	MDM2
Polygonum chinense	luteolin	APP
Polygonum chinense	luteolin	MMP1
Polygonum chinense	luteolin	PCNA
Polygonum chinense	luteolin	ERBB2
Polygonum chinense	luteolin	PPARG
Polygonum chinense	luteolin	HMOX1
Polygonum chinense	luteolin	CASP7
Polygonum chinense	luteolin	ICAM1
Polygonum chinense	luteolin	MCL1
Polygonum chinense	luteolin	BIRC5
Polygonum chinense	luteolin	IL2
Polygonum chinense	luteolin	CCNB1
Polygonum chinense	luteolin	TYR
Polygonum chinense	luteolin	IFNG
Polygonum chinense	luteolin	IL4
Polygonum chinense	luteolin	TOP2A
Polygonum chinense	luteolin	XIAP
Polygonum chinense	luteolin	SLC2A4
Polygonum chinense	luteolin	INSR
Polygonum chinense	luteolin	CD40LG
Polygonum chinense	luteolin	PTGES
Polygonum chinense	luteolin	NUF2
Polygonum chinense	luteolin	ADCY2
Polygonum chinense	luteolin	MET
Ecliptae Herba	acacetin	NOS2
Ecliptae Herba	acacetin	PTGS1
Ecliptae Herba	acacetin	AR
Ecliptae Herba	acacetin	PTGS2
Ecliptae Herba	acacetin	DPP4
Ecliptae Herba	acacetin	HSP90AA1
Ecliptae Herba	acacetin	CDK2
Ecliptae Herba	acacetin	PKIA
Ecliptae Herba	acacetin	PRSS1
Ecliptae Herba	acacetin	NCOA2
Ecliptae Herba	acacetin	NCOA1
Ecliptae Herba	acacetin	CALM2
Ecliptae Herba	acacetin	PIK3CG
Ecliptae Herba	acacetin	CHEK1
Ecliptae Herba	acacetin	ADRB2
Ecliptae Herba	acacetin	PDE3A
Ecliptae Herba	acacetin	RELA
Ecliptae Herba	acacetin	BCL2
Ecliptae Herba	acacetin	CDKN1A
Ecliptae Herba	acacetin	BAX
Ecliptae Herba	acacetin	CASP3
Ecliptae Herba	acacetin	TP53
Ecliptae Herba	acacetin	CASP8
Ecliptae Herba	acacetin	FASN

Ecliptae Herba	acacetin	FASLG
Ecliptae Herba	acacetin	CYP19A1
Ecliptae Herba	butin	PTGS1
Ecliptae Herba	butin	PTGS2
Ecliptae Herba	butin	RXRA
Ecliptae Herba	butin	HSP90AA1
Ecliptae Herba	butin	PIK3CG
Ecliptae Herba	butin	LACTBL1
Ecliptae Herba	butin	PKIA
Ecliptae Herba	1,3,8,9-tetrahydroxybenzofurano[3,2-c]chro	HSP90AA1
Ecliptae Herba	3'-O-Methylorobol	NOS2
Ecliptae Herba	3'-O-Methylorobol	PTGS1
Ecliptae Herba	3'-O-Methylorobol	ESR1
Ecliptae Herba	3'-O-Methylorobol	AR
Ecliptae Herba	3'-O-Methylorobol	PPARG
Ecliptae Herba	3'-O-Methylorobol	PTGS2
Ecliptae Herba	3'-O-Methylorobol	ESR2
Ecliptae Herba	3'-O-Methylorobol	MAPK14
Ecliptae Herba	3'-O-Methylorobol	GSK3B
Ecliptae Herba	3'-O-Methylorobol	HSP90AA1
Ecliptae Herba	3'-O-Methylorobol	CDK2
Ecliptae Herba	3'-O-Methylorobol	CHEK1
Ecliptae Herba	3'-O-Methylorobol	PKIA
Ecliptae Herba	3'-O-Methylorobol	PRSS1
Ecliptae Herba	3'-O-Methylorobol	PIM1
Ecliptae Herba	3'-O-Methylorobol	CCNA2
Ecliptae Herba	3'-O-Methylorobol	NCOA1
Ecliptae Herba	3'-O-Methylorobol	CALM2
Ecliptae Herba	Pratensein	NOS2
Ecliptae Herba	Pratensein	PTGS1
Ecliptae Herba	Pratensein	ESR1
Ecliptae Herba	Pratensein	AR
Ecliptae Herba	Pratensein	PPARG
Ecliptae Herba	Pratensein	PTGS2
Ecliptae Herba	Pratensein	ESR2
Ecliptae Herba	Pratensein	DPP4
Ecliptae Herba	Pratensein	MAPK14
Ecliptae Herba	Pratensein	GSK3B
Ecliptae Herba	Pratensein	HSP90AA1
Ecliptae Herba	Pratensein	CDK2
Ecliptae Herba	Pratensein	CHEK1
Ecliptae Herba	Pratensein	PKIA
Ecliptae Herba	Pratensein	PRSS1
Ecliptae Herba	Pratensein	PIM1
Ecliptae Herba	Pratensein	CCNA2
Ecliptae Herba	Pratensein	NCOA2
Ecliptae Herba	Pratensein	CALM2
Ecliptae Herba	demethylwedelolactone	PTGS2
Ecliptae Herba	demethylwedelolactone	GSK3B
Ecliptae Herba	wedelolactone	ESR1
Ecliptae Herba	wedelolactone	PPARG
Ecliptae Herba	wedelolactone	ESR2
Ecliptae Herba	wedelolactone	GSK3B
Ecliptae Herba	wedelolactone	HSP90AA1
Ecliptae Herba	wedelolactone	CDK2
Ecliptae Herba	wedelolactone	IKBKB
Ecliptae Herba	wedelolactone	AR
Ecliptae Herba	luteolin	PTGS1

Ecliptae Herba	luteolin	AR
Ecliptae Herba	luteolin	PTGS2
Ecliptae Herba	luteolin	HSP90AA1
Ecliptae Herba	luteolin	PRSS1
Ecliptae Herba	luteolin	NCOA2
Ecliptae Herba	luteolin	PKIA
Ecliptae Herba	luteolin	DPP4
Ecliptae Herba	luteolin	PIK3CG
Ecliptae Herba	luteolin	RELA
Ecliptae Herba	luteolin	EGFR
Ecliptae Herba	luteolin	AKT1
Ecliptae Herba	luteolin	CCND1
Ecliptae Herba	luteolin	BCL2L1
Ecliptae Herba	luteolin	CDKN1A
Ecliptae Herba	luteolin	CASP9
Ecliptae Herba	luteolin	MMP2
Ecliptae Herba	luteolin	MMP9
Ecliptae Herba	luteolin	MAPK1
Ecliptae Herba	luteolin	IL10
Ecliptae Herba	luteolin	RB1
Ecliptae Herba	luteolin	CDK4
Ecliptae Herba	luteolin	CD40LG
Ecliptae Herba	luteolin	JUN
Ecliptae Herba	luteolin	IL6
Ecliptae Herba	luteolin	CASP3
Ecliptae Herba	luteolin	TP53
Ecliptae Herba	luteolin	NFKBIA
Ecliptae Herba	luteolin	XDH
Ecliptae Herba	luteolin	TOP1
Ecliptae Herba	luteolin	MDM2
Ecliptae Herba	luteolin	APP
Ecliptae Herba	luteolin	MMP1
Ecliptae Herba	luteolin	PCNA
Ecliptae Herba	luteolin	ERBB2
Ecliptae Herba	luteolin	PPARG
Ecliptae Herba	luteolin	HMOX1
Ecliptae Herba	luteolin	CASP7
Ecliptae Herba	luteolin	ICAM1
Ecliptae Herba	luteolin	MCL1
Ecliptae Herba	luteolin	BIRC5
Ecliptae Herba	luteolin	IL2
Ecliptae Herba	luteolin	CCNB1
Ecliptae Herba	luteolin	TYR
Ecliptae Herba	luteolin	IFNG
Ecliptae Herba	luteolin	IL4
Ecliptae Herba	luteolin	TOP2A
Ecliptae Herba	luteolin	XIAP
Ecliptae Herba	luteolin	SLC2A4
Ecliptae Herba	luteolin	INSR
Ecliptae Herba	luteolin	CD40LG
Ecliptae Herba	luteolin	PTGES
Ecliptae Herba	luteolin	NUF2
Ecliptae Herba	luteolin	ADCY2
Ecliptae Herba	luteolin	MET
Perilla Frutescens	luteolin	PTGS1
Perilla Frutescens	luteolin	AR
Perilla Frutescens	luteolin	PTGS2
Perilla Frutescens	luteolin	HSP90AA1

Perilla Frutescens	luteolin	PRSS1
Perilla Frutescens	luteolin	NCOA2
Perilla Frutescens	luteolin	PKIA
Perilla Frutescens	luteolin	DPP4
Perilla Frutescens	luteolin	PIK3CG
Perilla Frutescens	luteolin	RELA
Perilla Frutescens	luteolin	EGFR
Perilla Frutescens	luteolin	AKT1
Perilla Frutescens	luteolin	CCND1
Perilla Frutescens	luteolin	BCL2L1
Perilla Frutescens	luteolin	CDKN1A
Perilla Frutescens	luteolin	CASP9
Perilla Frutescens	luteolin	MMP2
Perilla Frutescens	luteolin	MMP9
Perilla Frutescens	luteolin	MAPK1
Perilla Frutescens	luteolin	IL10
Perilla Frutescens	luteolin	RB1
Perilla Frutescens	luteolin	CDK4
Perilla Frutescens	luteolin	CD40LG
Perilla Frutescens	luteolin	JUN
Perilla Frutescens	luteolin	IL6
Perilla Frutescens	luteolin	CASP3
Perilla Frutescens	luteolin	TP53
Perilla Frutescens	luteolin	NFKBIA
Perilla Frutescens	luteolin	XDH
Perilla Frutescens	luteolin	TOP1
Perilla Frutescens	luteolin	MDM2
Perilla Frutescens	luteolin	APP
Perilla Frutescens	luteolin	MMP1
Perilla Frutescens	luteolin	PCNA
Perilla Frutescens	luteolin	ERBB2
Perilla Frutescens	luteolin	PPARG
Perilla Frutescens	luteolin	HMOX1
Perilla Frutescens	luteolin	CASP7
Perilla Frutescens	luteolin	ICAM1
Perilla Frutescens	luteolin	MCL1
Perilla Frutescens	luteolin	BIRC5
Perilla Frutescens	luteolin	IL2
Perilla Frutescens	luteolin	CCNB1
Perilla Frutescens	luteolin	TYR
Perilla Frutescens	luteolin	IFNG
Perilla Frutescens	luteolin	IL4
Perilla Frutescens	luteolin	TOP2A
Perilla Frutescens	luteolin	XIAP
Perilla Frutescens	luteolin	SLC2A4
Perilla Frutescens	luteolin	INSR
Perilla Frutescens	luteolin	CD40LG
Perilla Frutescens	luteolin	PTGES
Perilla Frutescens	luteolin	NUF2
Perilla Frutescens	luteolin	ADCY2
Perilla Frutescens	luteolin	MET
Isatidis Radix	acacetin	NOS2
Isatidis Radix	acacetin	PTGS1
Isatidis Radix	acacetin	AR
Isatidis Radix	acacetin	PTGS2
Isatidis Radix	acacetin	DPP4
Isatidis Radix	acacetin	HSP90AA1
Isatidis Radix	acacetin	CDK2

Isatidis Radix	acacetin	PKIA
Isatidis Radix	acacetin	PRSS1
Isatidis Radix	acacetin	NCOA2
Isatidis Radix	acacetin	NCOA1
Isatidis Radix	acacetin	CALM2
Isatidis Radix	acacetin	PIK3CG
Isatidis Radix	acacetin	CHEK1
Isatidis Radix	acacetin	ADRB2
Isatidis Radix	acacetin	PDE3A
Isatidis Radix	acacetin	RELA
Isatidis Radix	acacetin	BCL2
Isatidis Radix	acacetin	CDKN1A
Isatidis Radix	acacetin	BAX
Isatidis Radix	acacetin	CASP3
Isatidis Radix	acacetin	TP53
Isatidis Radix	acacetin	CASP8
Isatidis Radix	acacetin	FASN
Isatidis Radix	acacetin	FASLG
Isatidis Radix	acacetin	CYP19A1
Isatidis Radix	EUPATORIN	NOS2
Isatidis Radix	EUPATORIN	PTGS1
Isatidis Radix	EUPATORIN	F2
Isatidis Radix	EUPATORIN	SCN5A
Isatidis Radix	EUPATORIN	F10
Isatidis Radix	EUPATORIN	PTGS2
Isatidis Radix	EUPATORIN	F7
Isatidis Radix	EUPATORIN	TOP2A
Isatidis Radix	EUPATORIN	DPP4
Isatidis Radix	EUPATORIN	HSP90AA1
Isatidis Radix	EUPATORIN	PRSS1
Isatidis Radix	EUPATORIN	NCOA2
Isatidis Radix	EUPATORIN	NCOA1
Isatidis Radix	EUPATORIN	CALM2
Isatidis Radix	Dinatin	NOS2
Isatidis Radix	Dinatin	PTGS1
Isatidis Radix	Dinatin	PTGS2
Isatidis Radix	Dinatin	DPP4
Isatidis Radix	Dinatin	HSP90AA1
Isatidis Radix	Dinatin	PIK3CG
Isatidis Radix	Dinatin	PKIA
Isatidis Radix	Dinatin	PRSS1
Isatidis Radix	Dinatin	NCOA2
Isatidis Radix	Dinatin	NCOA1
Isatidis Radix	Dinatin	CALM2
Isatidis Radix	Dinatin	F2
Isatidis Radix	Dinatin	NOS3
Isatidis Radix	Dinatin	ACHE
Isatidis Radix	Dinatin	RHO
Isatidis Radix	quindoline	PTGS1
Isatidis Radix	quindoline	PTGS2
Isatidis Radix	quindoline	MAOB
Isatidis Radix	quindoline	PKIA
Isatidis Radix	quindoline	NCOA2
Isatidis Radix	quindoline	PKIA
Isatidis Radix	hydroxyindirubin	NOS2
Isatidis Radix	hydroxyindirubin	PTGS1
Isatidis Radix	hydroxyindirubin	ESR1
Isatidis Radix	hydroxyindirubin	AR

Isatidis Radix	hydroxyindirubin	PPARG
Isatidis Radix	hydroxyindirubin	PTGS2
Isatidis Radix	hydroxyindirubin	MAPK14
Isatidis Radix	hydroxyindirubin	GSK3B
Isatidis Radix	hydroxyindirubin	HSP90AA1
Isatidis Radix	hydroxyindirubin	CDK2
Isatidis Radix	hydroxyindirubin	PIK3CG
Isatidis Radix	hydroxyindirubin	PKIA
Isatidis Radix	hydroxyindirubin	PIM1
Isatidis Radix	hydroxyindirubin	CCNA2
Isatidis Radix	Indigo	PTGS1
Isatidis Radix	Indigo	PTGS2
Isatidis Radix	Indigo	RXRA
Isatidis Radix	Indigo	CDK2
Isatidis Radix	Indigo	PKIA
Isatidis Radix	Indigo	PIM1
Isatidis Radix	Indigo	CCNA2
Isatidis Radix	(2Z)-2-(2-oxoindolin-3-ylidene)indolin-3-o	NOS2
Isatidis Radix	(2Z)-2-(2-oxoindolin-3-ylidene)indolin-3-o	PTGS1
Isatidis Radix	(2Z)-2-(2-oxoindolin-3-ylidene)indolin-3-o	ESR1
Isatidis Radix	(2Z)-2-(2-oxoindolin-3-ylidene)indolin-3-o	AR
Isatidis Radix	(2Z)-2-(2-oxoindolin-3-ylidene)indolin-3-o	PTGS2
Isatidis Radix	(2Z)-2-(2-oxoindolin-3-ylidene)indolin-3-o	RXRA
Isatidis Radix	(2Z)-2-(2-oxoindolin-3-ylidene)indolin-3-o	GABRA1
Isatidis Radix	(2Z)-2-(2-oxoindolin-3-ylidene)indolin-3-o	MAPK14
Isatidis Radix	(2Z)-2-(2-oxoindolin-3-ylidene)indolin-3-o	GSK3B
Isatidis Radix	(2Z)-2-(2-oxoindolin-3-ylidene)indolin-3-o	HSP90AA1
Isatidis Radix	(2Z)-2-(2-oxoindolin-3-ylidene)indolin-3-o	CDK2
Isatidis Radix	(2Z)-2-(2-oxoindolin-3-ylidene)indolin-3-o	CHEK1
Isatidis Radix	(2Z)-2-(2-oxoindolin-3-ylidene)indolin-3-o	PKIA
Isatidis Radix	(2Z)-2-(2-oxoindolin-3-ylidene)indolin-3-o	PIM1
Isatidis Radix	(2Z)-2-(2-oxoindolin-3-ylidene)indolin-3-o	CCNA2
Isatidis Radix	2-(9-((3-methyl-2-oxopent-3-en-1-yl)oxy)-2	KCNH2
Isatidis Radix	2-(9-((3-methyl-2-oxopent-3-en-1-yl)oxy)-2	F10
Isatidis Radix	2-(9-((3-methyl-2-oxopent-3-en-1-yl)oxy)-2	PTGS2
Isatidis Radix	2-(9-((3-methyl-2-oxopent-3-en-1-yl)oxy)-2	TOP2A
Isatidis Radix	2-(9-((3-methyl-2-oxopent-3-en-1-yl)oxy)-2	HSP90AA1
Isatidis Radix	2-(9-((3-methyl-2-oxopent-3-en-1-yl)oxy)-2	PRSS1
Isatidis Radix	2-(9-((3-methyl-2-oxopent-3-en-1-yl)oxy)-2	NCOA2
Isatidis Radix	2-(9-((3-methyl-2-oxopent-3-en-1-yl)oxy)-2	KCNMA1
Isatidis Radix	DFV	PTGS1
Isatidis Radix	DFV	ESR1
Isatidis Radix	DFV	PTGS2
Isatidis Radix	DFV	RXRA
Isatidis Radix	DFV	ADRB2
Isatidis Radix	DFV	HSP90AA1
Isatidis Radix	DFV	PIK3CG
Isatidis Radix	DFV	PKIA
Isatidis Radix	DFV	LACTBL1
Isatidis Radix	DFV	MAOB
Isatidis Radix	DFV	SLC6A4
Isatidis Radix	DFV	PKIA
Isatidis Radix	(E)-2-[(3-indole)cyanomethylene-]-3-indoli	NOS2
Isatidis Radix	(E)-2-[(3-indole)cyanomethylene-]-3-indoli	PTGS1
Isatidis Radix	(E)-2-[(3-indole)cyanomethylene-]-3-indoli	ESR1
Isatidis Radix	(E)-2-[(3-indole)cyanomethylene-]-3-indoli	AR
Isatidis Radix	(E)-2-[(3-indole)cyanomethylene-]-3-indoli	PTGS2
Isatidis Radix	(E)-2-[(3-indole)cyanomethylene-]-3-indoli	RXRA

Isatidis Radix	(E)-2-[(3-indole)cyanomethylene-]-3-indoli	MAPK14
Isatidis Radix	(E)-2-[(3-indole)cyanomethylene-]-3-indoli	GSK3B
Isatidis Radix	(E)-2-[(3-indole)cyanomethylene-]-3-indoli	HSP90AA1
Isatidis Radix	(E)-2-[(3-indole)cyanomethylene-]-3-indoli	CDK2
Isatidis Radix	(E)-2-[(3-indole)cyanomethylene-]-3-indoli	PIK3CG
Isatidis Radix	(E)-2-[(3-indole)cyanomethylene-]-3-indoli	CHEK1
Isatidis Radix	(E)-2-[(3-indole)cyanomethylene-]-3-indoli	PKIA
Isatidis Radix	(E)-2-[(3-indole)cyanomethylene-]-3-indoli	PIM1
Isatidis Radix	(E)-2-[(3-indole)cyanomethylene-]-3-indoli	CCNA2
Isatidis Radix	neohesperidin_qt	PTGS1
Isatidis Radix	neohesperidin_qt	SCN5A
Isatidis Radix	neohesperidin_qt	PTGS2
Isatidis Radix	neohesperidin_qt	HSP90AA1
Isatidis Radix	neohesperidin_qt	PIK3CG
Isatidis Radix	neohesperidin_qt	PKIA
Isatidis Radix	neohesperidin_qt	CALM2
Isatidis Radix	Sinensetin	NOS2
Isatidis Radix	Sinensetin	F2
Isatidis Radix	Sinensetin	KCNH2
Isatidis Radix	Sinensetin	AR
Isatidis Radix	Sinensetin	SCN5A
Isatidis Radix	Sinensetin	F10
Isatidis Radix	Sinensetin	PTGS2
Isatidis Radix	Sinensetin	F7
Isatidis Radix	Sinensetin	ACHE
Isatidis Radix	Sinensetin	ADRB2
Isatidis Radix	Sinensetin	TOP2A
Isatidis Radix	Sinensetin	ESR2
Isatidis Radix	Sinensetin	DPP4
Isatidis Radix	Sinensetin	HSP90AA1
Isatidis Radix	Sinensetin	CHEK1
Isatidis Radix	Sinensetin	PRSS1
Isatidis Radix	Sinensetin	NCOA2
Isatidis Radix	Sinensetin	NCOA1
Isatidis Radix	Sinensetin	CALM2
Isatidis Radix	Sinensetin	PTGS1
Isatidis Radix	Sinensetin	ADRA1B
Isatidis Radix	6-(3-oxoindolin-2-ylidene)indolo[2,1-b]qui	PTGS1
Isatidis Radix	6-(3-oxoindolin-2-ylidene)indolo[2,1-b]qui	ESR1
Isatidis Radix	6-(3-oxoindolin-2-ylidene)indolo[2,1-b]qui	PTGS2
Isatidis Radix	6-(3-oxoindolin-2-ylidene)indolo[2,1-b]qui	KDR
Isatidis Radix	6-(3-oxoindolin-2-ylidene)indolo[2,1-b]qui	PRSS1
Isatidis Radix	(E)-3-(3,5-dimethoxy-4-hydroxy-benzylider	PTGS1
Isatidis Radix	(E)-3-(3,5-dimethoxy-4-hydroxy-benzylider	SCN5A
Isatidis Radix	(E)-3-(3,5-dimethoxy-4-hydroxy-benzylider	PTGS2
Isatidis Radix	(E)-3-(3,5-dimethoxy-4-hydroxy-benzylider	RXRA
Isatidis Radix	(E)-3-(3,5-dimethoxy-4-hydroxy-benzylider	PDE3A
Isatidis Radix	(E)-3-(3,5-dimethoxy-4-hydroxy-benzylider	GABRA1
Isatidis Radix	(E)-3-(3,5-dimethoxy-4-hydroxy-benzylider	HSP90AA1
Isatidis Radix	(E)-3-(3,5-dimethoxy-4-hydroxy-benzylider	PIK3CG
Isatidis Radix	(E)-3-(3,5-dimethoxy-4-hydroxy-benzylider	PKIA
Isatidis Radix	(E)-3-(3,5-dimethoxy-4-hydroxyb-enzylider	PTGS1
Isatidis Radix	(E)-3-(3,5-dimethoxy-4-hydroxyb-enzylider	CHRM1
Isatidis Radix	(E)-3-(3,5-dimethoxy-4-hydroxyb-enzylider	SCN5A
Isatidis Radix	(E)-3-(3,5-dimethoxy-4-hydroxyb-enzylider	PTGS2
Isatidis Radix	(E)-3-(3,5-dimethoxy-4-hydroxyb-enzylider	RXRA
Isatidis Radix	(E)-3-(3,5-dimethoxy-4-hydroxyb-enzylider	PDE3A
Isatidis Radix	(E)-3-(3,5-dimethoxy-4-hydroxyb-enzylider	PTPN1

Isatidis Radix	(E)-3-(3,5-dimethoxy-4-hydroxyb-enzyliden	ADRB2
Isatidis Radix	(E)-3-(3,5-dimethoxy-4-hydroxyb-enzyliden	GABRA1
Isatidis Radix	(E)-3-(3,5-dimethoxy-4-hydroxyb-enzyliden	HSP90AA1
Isatidis Radix	(E)-3-(3,5-dimethoxy-4-hydroxyb-enzyliden	PIK3CG
Isatidis Radix	(E)-3-(3,5-dimethoxy-4-hydroxyb-enzyliden	CHRNA7
Isatidis Radix	(E)-3-(3,5-dimethoxy-4-hydroxyb-enzyliden	PKIA
Isatidis Radix	3-[(3,5-dimethoxy-4-oxo-1-cyclohexa-2,5-d	PTGS1
Isatidis Radix	3-[(3,5-dimethoxy-4-oxo-1-cyclohexa-2,5-d	KCNH2
Isatidis Radix	3-[(3,5-dimethoxy-4-oxo-1-cyclohexa-2,5-d	SCN5A
Isatidis Radix	3-[(3,5-dimethoxy-4-oxo-1-cyclohexa-2,5-d	F10
Isatidis Radix	3-[(3,5-dimethoxy-4-oxo-1-cyclohexa-2,5-d	PTGS2
Isatidis Radix	3-[(3,5-dimethoxy-4-oxo-1-cyclohexa-2,5-d	F7
Isatidis Radix	3-[(3,5-dimethoxy-4-oxo-1-cyclohexa-2,5-d	HSP90AA1
Isatidis Radix	3-[(3,5-dimethoxy-4-oxo-1-cyclohexa-2,5-d	PRSS1
Chrysanthemi Flos	[(1S,5S,7S)-7-acetoxy-5-isopropenyl-2,8-di	NOS2
Chrysanthemi Flos	[(1S,5S,7S)-7-acetoxy-5-isopropenyl-2,8-di	F2
Chrysanthemi Flos	[(1S,5S,7S)-7-acetoxy-5-isopropenyl-2,8-di	AR
Chrysanthemi Flos	[(1S,5S,7S)-7-acetoxy-5-isopropenyl-2,8-di	PTGS2
Chrysanthemi Flos	[(1S,5S,7S)-7-acetoxy-5-isopropenyl-2,8-di	NOS3
Chrysanthemi Flos	[(1S,5S,7S)-7-acetoxy-5-isopropenyl-2,8-di	CA2
Chrysanthemi Flos	[(1S,5S,7S)-7-acetoxy-5-isopropenyl-2,8-di	DPP4
Chrysanthemi Flos	[(1S,5S,7S)-7-acetoxy-5-isopropenyl-2,8-di	NCOA2
Chrysanthemi Flos	acacetin	NOS2
Chrysanthemi Flos	acacetin	PTGS1
Chrysanthemi Flos	acacetin	AR
Chrysanthemi Flos	acacetin	PTGS2
Chrysanthemi Flos	acacetin	DPP4
Chrysanthemi Flos	acacetin	HSP90AA1
Chrysanthemi Flos	acacetin	CDK2
Chrysanthemi Flos	acacetin	PKIA
Chrysanthemi Flos	acacetin	PRSS1
Chrysanthemi Flos	acacetin	NCOA2
Chrysanthemi Flos	acacetin	NCOA1
Chrysanthemi Flos	acacetin	CALM2
Chrysanthemi Flos	acacetin	PIK3CG
Chrysanthemi Flos	acacetin	CHEK1
Chrysanthemi Flos	acacetin	ADRB2
Chrysanthemi Flos	acacetin	PDE3A
Chrysanthemi Flos	acacetin	RELA
Chrysanthemi Flos	acacetin	BCL2
Chrysanthemi Flos	acacetin	CDKN1A
Chrysanthemi Flos	acacetin	BAX
Chrysanthemi Flos	acacetin	CASP3
Chrysanthemi Flos	acacetin	TP53
Chrysanthemi Flos	acacetin	CASP8
Chrysanthemi Flos	acacetin	FASN
Chrysanthemi Flos	acacetin	FASLG
Chrysanthemi Flos	acacetin	CYP19A1
Chrysanthemi Flos	Chryseriol	NOS2
Chrysanthemi Flos	Chryseriol	PTGS1
Chrysanthemi Flos	Chryseriol	ESR1
Chrysanthemi Flos	Chryseriol	AR
Chrysanthemi Flos	Chryseriol	PPARG
Chrysanthemi Flos	Chryseriol	PTGS2
Chrysanthemi Flos	Chryseriol	DPP4
Chrysanthemi Flos	Chryseriol	MAPK14
Chrysanthemi Flos	Chryseriol	GSK3B
Chrysanthemi Flos	Chryseriol	HSP90AA1

Chrysanthemi Flos	Chryseriol	CDK2
Chrysanthemi Flos	Chryseriol	PIK3CG
Chrysanthemi Flos	Chryseriol	CHEK1
Chrysanthemi Flos	Chryseriol	PKIA
Chrysanthemi Flos	Chryseriol	PRSS1
Chrysanthemi Flos	Chryseriol	NCOA2
Chrysanthemi Flos	Chryseriol	NCOA1
Chrysanthemi Flos	Chryseriol	CALM2
Chrysanthemi Flos	isorhamnetin	NOS2
Chrysanthemi Flos	isorhamnetin	PTGS1
Chrysanthemi Flos	isorhamnetin	ESR1
Chrysanthemi Flos	isorhamnetin	AR
Chrysanthemi Flos	isorhamnetin	PPARG
Chrysanthemi Flos	isorhamnetin	PTGS2
Chrysanthemi Flos	isorhamnetin	PTPN1
Chrysanthemi Flos	isorhamnetin	ESR2
Chrysanthemi Flos	isorhamnetin	DPP4
Chrysanthemi Flos	isorhamnetin	MAPK14
Chrysanthemi Flos	isorhamnetin	GSK3B
Chrysanthemi Flos	isorhamnetin	HSP90AA1
Chrysanthemi Flos	isorhamnetin	CDK2
Chrysanthemi Flos	isorhamnetin	PIK3CG
Chrysanthemi Flos	isorhamnetin	PKIA
Chrysanthemi Flos	isorhamnetin	PRSS1
Chrysanthemi Flos	isorhamnetin	PIM1
Chrysanthemi Flos	isorhamnetin	CCNA2
Chrysanthemi Flos	isorhamnetin	NCOA2
Chrysanthemi Flos	isorhamnetin	CALM2
Chrysanthemi Flos	isorhamnetin	PYGM
Chrysanthemi Flos	isorhamnetin	PPARD
Chrysanthemi Flos	isorhamnetin	CHEK1
Chrysanthemi Flos	isorhamnetin	AKR1B1
Chrysanthemi Flos	isorhamnetin	NCOA1
Chrysanthemi Flos	isorhamnetin	F7
Chrysanthemi Flos	isorhamnetin	F2
Chrysanthemi Flos	isorhamnetin	NOS3
Chrysanthemi Flos	isorhamnetin	ACHE
Chrysanthemi Flos	isorhamnetin	GABRA1
Chrysanthemi Flos	isorhamnetin	MAOB
Chrysanthemi Flos	isorhamnetin	GRIA2
Chrysanthemi Flos	isorhamnetin	RELA
Chrysanthemi Flos	isorhamnetin	XDH
Chrysanthemi Flos	isorhamnetin	NCF1
Chrysanthemi Flos	isorhamnetin	OLR1
Chrysanthemi Flos	kaempferol	NOS2
Chrysanthemi Flos	kaempferol	PTGS1
Chrysanthemi Flos	kaempferol	AR
Chrysanthemi Flos	kaempferol	PPARG
Chrysanthemi Flos	kaempferol	PTGS2
Chrysanthemi Flos	kaempferol	HSP90AA1
Chrysanthemi Flos	kaempferol	PIK3CG
Chrysanthemi Flos	kaempferol	PKIA
Chrysanthemi Flos	kaempferol	NCOA2
Chrysanthemi Flos	kaempferol	DPP4
Chrysanthemi Flos	kaempferol	PRSS1
Chrysanthemi Flos	kaempferol	PGR
Chrysanthemi Flos	kaempferol	F2
Chrysanthemi Flos	kaempferol	CHRM1

Chrysanthemi Flos	kaempferol	NOS3
Chrysanthemi Flos	kaempferol	GABRA2
Chrysanthemi Flos	kaempferol	ACHE
Chrysanthemi Flos	kaempferol	SLC6A2
Chrysanthemi Flos	kaempferol	CHRM2
Chrysanthemi Flos	kaempferol	ADRA1B
Chrysanthemi Flos	kaempferol	GABRA1
Chrysanthemi Flos	kaempferol	TOP2A
Chrysanthemi Flos	kaempferol	F7
Chrysanthemi Flos	kaempferol	CALM2
Chrysanthemi Flos	kaempferol	RELA
Chrysanthemi Flos	kaempferol	IKBKB
Chrysanthemi Flos	kaempferol	AKT1
Chrysanthemi Flos	kaempferol	BCL2
Chrysanthemi Flos	kaempferol	BAX
Chrysanthemi Flos	kaempferol	CD40LG
Chrysanthemi Flos	kaempferol	JUN
Chrysanthemi Flos	kaempferol	AHSA1
Chrysanthemi Flos	kaempferol	CASP3
Chrysanthemi Flos	kaempferol	MAPK8
Chrysanthemi Flos	kaempferol	XDH
Chrysanthemi Flos	kaempferol	MMP1
Chrysanthemi Flos	kaempferol	STAT1
Chrysanthemi Flos	kaempferol	CDK1
Chrysanthemi Flos	kaempferol	PPARG
Chrysanthemi Flos	kaempferol	HMOX1
Chrysanthemi Flos	kaempferol	CYP3A4
Chrysanthemi Flos	kaempferol	CYP1A1
Chrysanthemi Flos	kaempferol	ICAM1
Chrysanthemi Flos	kaempferol	SELE
Chrysanthemi Flos	kaempferol	VCAM1
Chrysanthemi Flos	kaempferol	NR1I2
Chrysanthemi Flos	kaempferol	CYP1B1
Chrysanthemi Flos	kaempferol	ALOX5
Chrysanthemi Flos	kaempferol	HAS2
Chrysanthemi Flos	kaempferol	AHR
Chrysanthemi Flos	kaempferol	PSMD3
Chrysanthemi Flos	kaempferol	SLC2A4
Chrysanthemi Flos	kaempferol	NR1I3
Chrysanthemi Flos	kaempferol	INSR
Chrysanthemi Flos	kaempferol	DIO1
Chrysanthemi Flos	kaempferol	PPP3CA
Chrysanthemi Flos	kaempferol	GSTM1
Chrysanthemi Flos	kaempferol	GSTM2
Chrysanthemi Flos	kaempferol	AKR1C3
Chrysanthemi Flos	kaempferol	SLPI
Chrysanthemi Flos	5,7-dihydroxy-2-(3-hydroxy-4-methoxyphe	PTGS1
Chrysanthemi Flos	5,7-dihydroxy-2-(3-hydroxy-4-methoxyphe	PTGS2
Chrysanthemi Flos	5,7-dihydroxy-2-(3-hydroxy-4-methoxyphe	HSP90AA1
Chrysanthemi Flos	5,7-dihydroxy-2-(3-hydroxy-4-methoxyphe	PKIA
Chrysanthemi Flos	5,7-dihydroxy-2-(3-hydroxy-4-methoxyphe	CALM2
Chrysanthemi Flos	5,7-dihydroxy-2-(3-hydroxy-4-methoxyphe	SCN5A
Chrysanthemi Flos	5,7-dihydroxy-2-(3-hydroxy-4-methoxyphe	PIK3CG
Chrysanthemi Flos	5,7-dihydroxy-2-(3-hydroxy-4-methoxyphe	NCOA2
Chrysanthemi Flos	5,7-dihydroxy-2-(3-hydroxy-4-methoxyphe	NCOA1
Chrysanthemi Flos	5,7-dihydroxy-2-(3-hydroxy-4-methoxyphe	TOP2A
Chrysanthemi Flos	luteolin	PTGS1
Chrysanthemi Flos	luteolin	AR

Chrysanthemi Flos	luteolin	PTGS2
Chrysanthemi Flos	luteolin	HSP90AA1
Chrysanthemi Flos	luteolin	PRSS1
Chrysanthemi Flos	luteolin	NCOA2
Chrysanthemi Flos	luteolin	PKIA
Chrysanthemi Flos	luteolin	DPP4
Chrysanthemi Flos	luteolin	PIK3CG
Chrysanthemi Flos	luteolin	RELA
Chrysanthemi Flos	luteolin	EGFR
Chrysanthemi Flos	luteolin	AKT1
Chrysanthemi Flos	luteolin	CCND1
Chrysanthemi Flos	luteolin	BCL2L1
Chrysanthemi Flos	luteolin	CDKN1A
Chrysanthemi Flos	luteolin	CASP9
Chrysanthemi Flos	luteolin	MMP2
Chrysanthemi Flos	luteolin	MMP9
Chrysanthemi Flos	luteolin	MAPK1
Chrysanthemi Flos	luteolin	IL10
Chrysanthemi Flos	luteolin	RB1
Chrysanthemi Flos	luteolin	CDK4
Chrysanthemi Flos	luteolin	CD40LG
Chrysanthemi Flos	luteolin	JUN
Chrysanthemi Flos	luteolin	IL6
Chrysanthemi Flos	luteolin	CASP3
Chrysanthemi Flos	luteolin	TP53
Chrysanthemi Flos	luteolin	NFKBIA
Chrysanthemi Flos	luteolin	XDH
Chrysanthemi Flos	luteolin	TOP1
Chrysanthemi Flos	luteolin	MDM2
Chrysanthemi Flos	luteolin	APP
Chrysanthemi Flos	luteolin	MMP1
Chrysanthemi Flos	luteolin	PCNA
Chrysanthemi Flos	luteolin	ERBB2
Chrysanthemi Flos	luteolin	PPARG
Chrysanthemi Flos	luteolin	HMOX1
Chrysanthemi Flos	luteolin	CASP7
Chrysanthemi Flos	luteolin	ICAM1
Chrysanthemi Flos	luteolin	MCL1
Chrysanthemi Flos	luteolin	BIRC5
Chrysanthemi Flos	luteolin	IL2
Chrysanthemi Flos	luteolin	CCNB1
Chrysanthemi Flos	luteolin	TYR
Chrysanthemi Flos	luteolin	IFNG
Chrysanthemi Flos	luteolin	IL4
Chrysanthemi Flos	luteolin	TOP2A
Chrysanthemi Flos	luteolin	XIAP
Chrysanthemi Flos	luteolin	SLC2A4
Chrysanthemi Flos	luteolin	INSR
Chrysanthemi Flos	luteolin	CD40LG
Chrysanthemi Flos	luteolin	PTGES
Chrysanthemi Flos	luteolin	NUF2
Chrysanthemi Flos	luteolin	ADCY2
Chrysanthemi Flos	luteolin	MET
Chrysanthemi Flos	EUPATORIN	NOS2
Chrysanthemi Flos	EUPATORIN	PTGS1
Chrysanthemi Flos	EUPATORIN	F2
Chrysanthemi Flos	EUPATORIN	SCN5A
Chrysanthemi Flos	EUPATORIN	F10

Chrysanthemi Flos	EUPATORIN	PTGS2
Chrysanthemi Flos	EUPATORIN	F7
Chrysanthemi Flos	EUPATORIN	TOP2A
Chrysanthemi Flos	EUPATORIN	DPP4
Chrysanthemi Flos	EUPATORIN	HSP90AA1
Chrysanthemi Flos	EUPATORIN	PRSS1
Chrysanthemi Flos	EUPATORIN	NCOA2
Chrysanthemi Flos	EUPATORIN	NCOA1
Chrysanthemi Flos	EUPATORIN	CALM2
Chrysanthemi Flos	Diosmetin	NOS2
Chrysanthemi Flos	Diosmetin	PTGS1
Chrysanthemi Flos	Diosmetin	PTGS2
Chrysanthemi Flos	Diosmetin	DPP4
Chrysanthemi Flos	Diosmetin	HSP90AA1
Chrysanthemi Flos	Diosmetin	PKIA
Chrysanthemi Flos	Diosmetin	PRSS1
Chrysanthemi Flos	Diosmetin	NCOA2
Chrysanthemi Flos	Diosmetin	NCOA1
Chrysanthemi Flos	Diosmetin	CALM2
Chrysanthemi Flos	naringenin	PTGS1
Chrysanthemi Flos	naringenin	ESR1
Chrysanthemi Flos	naringenin	PTGS2
Chrysanthemi Flos	naringenin	HSP90AA1
Chrysanthemi Flos	naringenin	LACTBL1
Chrysanthemi Flos	naringenin	PKIA
Chrysanthemi Flos	naringenin	PIK3CG
Chrysanthemi Flos	naringenin	RELA
Chrysanthemi Flos	naringenin	AKT1
Chrysanthemi Flos	naringenin	BCL2
Chrysanthemi Flos	naringenin	MAPK3
Chrysanthemi Flos	naringenin	MAPK1
Chrysanthemi Flos	naringenin	CASP3
Chrysanthemi Flos	naringenin	FASN
Chrysanthemi Flos	naringenin	LDLR
Chrysanthemi Flos	naringenin	BAD
Chrysanthemi Flos	naringenin	SOD1
Chrysanthemi Flos	naringenin	PPARG
Chrysanthemi Flos	naringenin	MTTP
Chrysanthemi Flos	naringenin	APOB
Chrysanthemi Flos	naringenin	PLB1
Chrysanthemi Flos	naringenin	HMGCR
Chrysanthemi Flos	naringenin	CYP19A1
Chrysanthemi Flos	naringenin	UGT1A8
Chrysanthemi Flos	naringenin	PPARA
Chrysanthemi Flos	naringenin	SREBF1
Chrysanthemi Flos	naringenin	GSR
Chrysanthemi Flos	naringenin	ABCC1
Chrysanthemi Flos	naringenin	ADIPOQ
Chrysanthemi Flos	naringenin	SOAT2
Chrysanthemi Flos	naringenin	AKR1C1
Chrysanthemi Flos	naringenin	GOT1
Chrysanthemi Flos	naringenin	ABAT
Chrysanthemi Flos	naringenin	CES1
Chrysanthemi Flos	naringenin	SOAT1
Chrysanthemi Flos	Artemetin	NOS2
Chrysanthemi Flos	Artemetin	F2
Chrysanthemi Flos	Artemetin	KCNH2
Chrysanthemi Flos	Artemetin	ESR1

Chrysanthemi Flos	Artemetin	AR
Chrysanthemi Flos	Artemetin	SCN5A
Chrysanthemi Flos	Artemetin	PPARG
Chrysanthemi Flos	Artemetin	F10
Chrysanthemi Flos	Artemetin	PTGS2
Chrysanthemi Flos	Artemetin	F7
Chrysanthemi Flos	Artemetin	PTPN1
Chrysanthemi Flos	Artemetin	TOP2A
Chrysanthemi Flos	Artemetin	ESR2
Chrysanthemi Flos	Artemetin	DPP4
Chrysanthemi Flos	Artemetin	MAPK14
Chrysanthemi Flos	Artemetin	GSK3B
Chrysanthemi Flos	Artemetin	HSP90AA1
Chrysanthemi Flos	Artemetin	CDK2
Chrysanthemi Flos	Artemetin	CHEK1
Chrysanthemi Flos	Artemetin	PRSS1
Chrysanthemi Flos	Artemetin	NCOA2
Chrysanthemi Flos	Artemetin	KCNMA1
Chrysanthemi Flos	Artemetin	CALM2
licorice	Jaranol	NOS2
licorice	Jaranol	PTGS1
licorice	Jaranol	AR
licorice	Jaranol	SCN5A
licorice	Jaranol	PTGS2
licorice	Jaranol	ESR2
licorice	Jaranol	DPP4
licorice	Jaranol	HSP90AA1
licorice	Jaranol	CDK2
licorice	Jaranol	CHEK1
licorice	Jaranol	PRSS1
licorice	Jaranol	NCOA2
licorice	Jaranol	CALM2
licorice	isorhamnetin	NOS2
licorice	isorhamnetin	PTGS1
licorice	isorhamnetin	ESR1
licorice	isorhamnetin	AR
licorice	isorhamnetin	PPARG
licorice	isorhamnetin	PTGS2
licorice	isorhamnetin	PTPN1
licorice	isorhamnetin	ESR2
licorice	isorhamnetin	DPP4
licorice	isorhamnetin	MAPK14
licorice	isorhamnetin	GSK3B
licorice	isorhamnetin	HSP90AA1
licorice	isorhamnetin	CDK2
licorice	isorhamnetin	PIK3CG
licorice	isorhamnetin	PKIA
licorice	isorhamnetin	PRSS1
licorice	isorhamnetin	PIM1
licorice	isorhamnetin	CCNA2
licorice	isorhamnetin	NCOA2
licorice	isorhamnetin	CALM2
licorice	isorhamnetin	PYGM
licorice	isorhamnetin	PPARD
licorice	isorhamnetin	CHEK1
licorice	isorhamnetin	AKR1B1
licorice	isorhamnetin	NCOA1
licorice	isorhamnetin	F7

licorice	isorhamnetin	F2
licorice	isorhamnetin	NOS3
licorice	isorhamnetin	ACHE
licorice	isorhamnetin	GABRA1
licorice	isorhamnetin	MAOB
licorice	isorhamnetin	GRIA2
licorice	isorhamnetin	RELA
licorice	isorhamnetin	XDH
licorice	isorhamnetin	NCF1
licorice	isorhamnetin	OLR1
licorice	formononetin	NOS2
licorice	formononetin	PTGS1
licorice	formononetin	CHRM1
licorice	formononetin	ESR1
licorice	formononetin	AR
licorice	formononetin	PPARG
licorice	formononetin	PTGS2
licorice	formononetin	RXRA
licorice	formononetin	PDE3A
licorice	formononetin	ADRA1D
licorice	formononetin	SLC6A3
licorice	formononetin	ADRB2
licorice	formononetin	SLC6A4
licorice	formononetin	ESR2
licorice	formononetin	DPP4
licorice	formononetin	MAPK14
licorice	formononetin	GSK3B
licorice	formononetin	HSP90AA1
licorice	formononetin	CDK2
licorice	formononetin	MAOB
licorice	formononetin	CHEK1
licorice	formononetin	PKIA
licorice	formononetin	PRSS1
licorice	formononetin	PIM1
licorice	formononetin	CCNA2
licorice	formononetin	CALM2
licorice	formononetin	PKIA
licorice	formononetin	F2
licorice	formononetin	NOS3
licorice	formononetin	ACHE
licorice	formononetin	LACTBL1
licorice	formononetin	JUN
licorice	formononetin	PPARG
licorice	formononetin	IL4
licorice	formononetin	SIRT1
licorice	formononetin	ATP5B
licorice	formononetin	MT-ND6
licorice	formononetin	HSD3B2
licorice	formononetin	HSD3B1
licorice	Calycosin	NOS2
licorice	Calycosin	PTGS1
licorice	Calycosin	ESR1
licorice	Calycosin	AR
licorice	Calycosin	PPARG
licorice	Calycosin	PTGS2
licorice	Calycosin	RXRA
licorice	Calycosin	PDE3A
licorice	Calycosin	ESR2

licorice	Calycosin	DPP4
licorice	Calycosin	MAPK14
licorice	Calycosin	GSK3B
licorice	Calycosin	HSP90AA1
licorice	Calycosin	CDK2
licorice	Calycosin	CHEK1
licorice	Calycosin	PKIA
licorice	Calycosin	PRSS1
licorice	Calycosin	PIM1
licorice	Calycosin	CCNA2
licorice	Calycosin	NCOA2
licorice	Calycosin	CALM2
licorice	Calycosin	ADRB2
licorice	kaempferol	NOS2
licorice	kaempferol	PTGS1
licorice	kaempferol	AR
licorice	kaempferol	PPARG
licorice	kaempferol	PTGS2
licorice	kaempferol	HSP90AA1
licorice	kaempferol	PIK3CG
licorice	kaempferol	PKIA
licorice	kaempferol	NCOA2
licorice	kaempferol	DPP4
licorice	kaempferol	PRSS1
licorice	kaempferol	PGR
licorice	kaempferol	F2
licorice	kaempferol	CHRM1
licorice	kaempferol	NOS3
licorice	kaempferol	GABRA2
licorice	kaempferol	ACHE
licorice	kaempferol	SLC6A2
licorice	kaempferol	CHRM2
licorice	kaempferol	ADRA1B
licorice	kaempferol	GABRA1
licorice	kaempferol	TOP2A
licorice	kaempferol	F7
licorice	kaempferol	CALM2
licorice	kaempferol	RELA
licorice	kaempferol	IKBKB
licorice	kaempferol	AKT1
licorice	kaempferol	BCL2
licorice	kaempferol	BAX
licorice	kaempferol	CD40LG
licorice	kaempferol	JUN
licorice	kaempferol	AHSA1
licorice	kaempferol	CASP3
licorice	kaempferol	MAPK8
licorice	kaempferol	XDH
licorice	kaempferol	MMP1
licorice	kaempferol	STAT1
licorice	kaempferol	CDK1
licorice	kaempferol	PPARG
licorice	kaempferol	HMOX1
licorice	kaempferol	CYP3A4
licorice	kaempferol	CYP1A1
licorice	kaempferol	ICAM1
licorice	kaempferol	SELE
licorice	kaempferol	VCAM1

licorice	kaempferol	NR1I2
licorice	kaempferol	CYP1B1
licorice	kaempferol	ALOX5
licorice	kaempferol	HAS2
licorice	kaempferol	AHR
licorice	kaempferol	PSMD3
licorice	kaempferol	SLC2A4
licorice	kaempferol	NR1I3
licorice	kaempferol	INSR
licorice	kaempferol	DIO1
licorice	kaempferol	PPP3CA
licorice	kaempferol	GSTM1
licorice	kaempferol	GSTM2
licorice	kaempferol	AKR1C3
licorice	kaempferol	SLPI
licorice	licochalcone a	NOS2
licorice	licochalcone a	PTGS1
licorice	licochalcone a	CHRM1
licorice	licochalcone a	ESR1
licorice	licochalcone a	AR
licorice	licochalcone a	SCN5A
licorice	licochalcone a	PPARG
licorice	licochalcone a	F10
licorice	licochalcone a	PTGS2
licorice	licochalcone a	CA2
licorice	licochalcone a	ADRA1B
licorice	licochalcone a	SLC6A3
licorice	licochalcone a	ESR2
licorice	licochalcone a	MAPK14
licorice	licochalcone a	GSK3B
licorice	licochalcone a	HSP90AA1
licorice	licochalcone a	CDK2
licorice	licochalcone a	CHEK1
licorice	licochalcone a	PIM1
licorice	licochalcone a	CCNA2
licorice	licochalcone a	CALM2
licorice	licochalcone a	ADRB2
licorice	licochalcone a	NCOA2
licorice	licochalcone a	RELA
licorice	licochalcone a	STAT3
licorice	licochalcone a	CCND1
licorice	licochalcone a	BCL2
licorice	licochalcone a	EIF6
licorice	licochalcone a	MAPK1
licorice	licochalcone a	RB1
licorice	licochalcone a	CDK4
licorice	licochalcone a	FOSL2
licorice	Inermine	PTGS1
licorice	Inermine	CHRM3
licorice	Inermine	SCN5A
licorice	Inermine	PTGS2
licorice	Inermine	RXRA
licorice	Inermine	ADRA1B
licorice	Inermine	ADRA1D
licorice	Inermine	PIK3CG
licorice	Inermine	PKIA
licorice	Inermine	PRSS1
licorice	Inermine	CALM2

licorice	Inermine	CHRM1
licorice	Inermine	ADRB2
licorice	Inermine	OPRM1
licorice	Inermine	HSP90AA1
licorice	DFV	PTGS1
licorice	DFV	ESR1
licorice	DFV	PTGS2
licorice	DFV	RXRA
licorice	DFV	ADRB2
licorice	DFV	HSP90AA1
licorice	DFV	PIK3CG
licorice	DFV	PKIA
licorice	DFV	LACTBL1
licorice	DFV	MAOB
licorice	DFV	SLC6A4
licorice	DFV	PKIA
licorice	Glycyrol	NOS2
licorice	Glycyrol	ESR1
licorice	Glycyrol	PPARG
licorice	Glycyrol	PTGS2
licorice	Glycyrol	KDR
licorice	Glycyrol	MAPK14
licorice	Glycyrol	GSK3B
licorice	Glycyrol	CHEK1
licorice	Glycyrol	PIM1
licorice	Glycyrol	CCNA2
licorice	Glycyrol	F2
licorice	Medicarpin	NOS2
licorice	Medicarpin	PTGS1
licorice	Medicarpin	DRD1
licorice	Medicarpin	CHRM3
licorice	Medicarpin	CHRM1
licorice	Medicarpin	ESR1
licorice	Medicarpin	SCN5A
licorice	Medicarpin	CHRM5
licorice	Medicarpin	PTGS2
licorice	Medicarpin	CHRM4
licorice	Medicarpin	RXRA
licorice	Medicarpin	HTR2A
licorice	Medicarpin	ADRA1D
licorice	Medicarpin	CHRM2
licorice	Medicarpin	ADRA1B
licorice	Medicarpin	SLC6A3
licorice	Medicarpin	ADRB2
licorice	Medicarpin	SLC6A4
licorice	Medicarpin	OPRM1
licorice	Medicarpin	ESR2
licorice	Medicarpin	DPP4
licorice	Medicarpin	MAPK10
licorice	Medicarpin	HSP90AA1
licorice	Medicarpin	CDK2
licorice	Medicarpin	PIK3CG
licorice	Medicarpin	CHRNA7
licorice	Medicarpin	PKIA
licorice	Medicarpin	PRSS1
licorice	Medicarpin	PIM1
licorice	Medicarpin	CCNA2
licorice	Medicarpin	CALM2

licorice	Medicarpin	OPRD1
licorice	Medicarpin	PDE3A
licorice	Medicarpin	ADRA1D
licorice	Lupiwighteone	NOS2
licorice	Lupiwighteone	F2
licorice	Lupiwighteone	ESR1
licorice	Lupiwighteone	AR
licorice	Lupiwighteone	SCN5A
licorice	Lupiwighteone	PPARG
licorice	Lupiwighteone	F10
licorice	Lupiwighteone	PTGS2
licorice	Lupiwighteone	TOP2A
licorice	Lupiwighteone	ESR2
licorice	Lupiwighteone	DPP4
licorice	Lupiwighteone	MAPK14
licorice	Lupiwighteone	GSK3B
licorice	Lupiwighteone	HSP90AA1
licorice	Lupiwighteone	CDK2
licorice	Lupiwighteone	CHEK1
licorice	Lupiwighteone	PRSS1
licorice	Lupiwighteone	PIM1
licorice	Lupiwighteone	CCNA2
licorice	Lupiwighteone	NCOA2
licorice	Lupiwighteone	CALM2
licorice	7-Methoxy-2-methyl isoflavone	NOS2
licorice	7-Methoxy-2-methyl isoflavone	PTGS1
licorice	7-Methoxy-2-methyl isoflavone	DRD1
licorice	7-Methoxy-2-methyl isoflavone	CHRM3
licorice	7-Methoxy-2-methyl isoflavone	F2
licorice	7-Methoxy-2-methyl isoflavone	CHRM1
licorice	7-Methoxy-2-methyl isoflavone	ESR1
licorice	7-Methoxy-2-methyl isoflavone	AR
licorice	7-Methoxy-2-methyl isoflavone	ADRB1
licorice	7-Methoxy-2-methyl isoflavone	SCN5A
licorice	7-Methoxy-2-methyl isoflavone	PPARG
licorice	7-Methoxy-2-methyl isoflavone	PTGS2
licorice	7-Methoxy-2-methyl isoflavone	RXRA
licorice	7-Methoxy-2-methyl isoflavone	ACHE
licorice	7-Methoxy-2-methyl isoflavone	PDE3A
licorice	7-Methoxy-2-methyl isoflavone	ADRA1B
licorice	7-Methoxy-2-methyl isoflavone	SLC6A3
licorice	7-Methoxy-2-methyl isoflavone	ADRB2
licorice	7-Methoxy-2-methyl isoflavone	ADRA1D
licorice	7-Methoxy-2-methyl isoflavone	SLC6A4
licorice	7-Methoxy-2-methyl isoflavone	ESR2
licorice	7-Methoxy-2-methyl isoflavone	GABRA1
licorice	7-Methoxy-2-methyl isoflavone	DPP4
licorice	7-Methoxy-2-methyl isoflavone	MAPK14
licorice	7-Methoxy-2-methyl isoflavone	GSK3B
licorice	7-Methoxy-2-methyl isoflavone	HSP90AA1
licorice	7-Methoxy-2-methyl isoflavone	CDK2
licorice	7-Methoxy-2-methyl isoflavone	LTA4H
licorice	7-Methoxy-2-methyl isoflavone	MAOB
licorice	7-Methoxy-2-methyl isoflavone	CHRNA7
licorice	7-Methoxy-2-methyl isoflavone	CHEK1
licorice	7-Methoxy-2-methyl isoflavone	PKIA
licorice	7-Methoxy-2-methyl isoflavone	PRSS1
licorice	7-Methoxy-2-methyl isoflavone	PIM1

licorice	7-Methoxy-2-methyl isoflavone	CCNA2
licorice	7-Methoxy-2-methyl isoflavone	NCOA1
licorice	7-Methoxy-2-methyl isoflavone	PKIA
licorice	7-Methoxy-2-methyl isoflavone	CALM2
licorice	7-Methoxy-2-methyl isoflavone	CHRM5
licorice	7-Methoxy-2-methyl isoflavone	NOS3
licorice	7-Methoxy-2-methyl isoflavone	OPRM1
licorice	7-Methoxy-2-methyl isoflavone	NCOA2
licorice	naringenin	PTGS1
licorice	naringenin	ESR1
licorice	naringenin	PTGS2
licorice	naringenin	HSP90AA1
licorice	naringenin	LACTBL1
licorice	naringenin	PKIA
licorice	naringenin	PIK3CG
licorice	naringenin	RELA
licorice	naringenin	AKT1
licorice	naringenin	BCL2
licorice	naringenin	MAPK3
licorice	naringenin	MAPK1
licorice	naringenin	CASP3
licorice	naringenin	FASN
licorice	naringenin	LDLR
licorice	naringenin	BAD
licorice	naringenin	SOD1
licorice	naringenin	PPARG
licorice	naringenin	MTTP
licorice	naringenin	APOB
licorice	naringenin	PLB1
licorice	naringenin	HMGCR
licorice	naringenin	CYP19A1
licorice	naringenin	UGT1A8
licorice	naringenin	PPARA
licorice	naringenin	SREBF1
licorice	naringenin	GSR
licorice	naringenin	ABCC1
licorice	naringenin	ADIPOQ
licorice	naringenin	SOAT2
licorice	naringenin	AKR1C1
licorice	naringenin	GOT1
licorice	naringenin	ABAT
licorice	naringenin	CES1
licorice	naringenin	SOAT1
licorice	glyasperin B	NOS2
licorice	glyasperin B	F2
licorice	glyasperin B	ESR1
licorice	glyasperin B	AR
licorice	glyasperin B	PPARG
licorice	glyasperin B	F10
licorice	glyasperin B	PTGS2
licorice	glyasperin B	F7
licorice	glyasperin B	KDR
licorice	glyasperin B	ACHE
licorice	glyasperin B	TOP2A
licorice	glyasperin B	ESR2
licorice	glyasperin B	DPP4
licorice	glyasperin B	GSK3B
licorice	glyasperin B	HSP90AA1

licorice	glyasperin B	CDK2
licorice	glyasperin B	PRSS1
licorice	glyasperin B	PIM1
licorice	glyasperin B	CCNA2
licorice	glyasperin B	NCOA2
licorice	glyasperin B	CALM2
licorice	glyasperin F	NOS2
licorice	glyasperin F	PTGS1
licorice	glyasperin F	ESR1
licorice	glyasperin F	AR
licorice	glyasperin F	SCN5A
licorice	glyasperin F	PPARG
licorice	glyasperin F	F10
licorice	glyasperin F	PTGS2
licorice	glyasperin F	TOP2A
licorice	glyasperin F	ESR2
licorice	glyasperin F	MAPK14
licorice	glyasperin F	GSK3B
licorice	glyasperin F	HSP90AA1
licorice	glyasperin F	CDK2
licorice	glyasperin F	PRSS1
licorice	glyasperin F	PIM1
licorice	glyasperin F	CCNA2
licorice	glyasperin F	CALM2
licorice	Isotrifoliol	NOS2
licorice	Isotrifoliol	ESR1
licorice	Isotrifoliol	AR
licorice	Isotrifoliol	PTGS2
licorice	Isotrifoliol	ESR2
licorice	Isotrifoliol	MAPK14
licorice	Isotrifoliol	GSK3B
licorice	Isotrifoliol	HSP90AA1
licorice	Isotrifoliol	CDK2
licorice	Isotrifoliol	PIK3CG
licorice	Isotrifoliol	CHEK1
licorice	Isotrifoliol	PKIA
licorice	Isotrifoliol	PIM1
licorice	Isotrifoliol	CCNA2
licorice	(E)-1-(2,4-dihydroxyphenyl)-3-(2,2-dimeth	NOS2
licorice	(E)-1-(2,4-dihydroxyphenyl)-3-(2,2-dimeth	PTGS1
licorice	(E)-1-(2,4-dihydroxyphenyl)-3-(2,2-dimeth	ESR1
licorice	(E)-1-(2,4-dihydroxyphenyl)-3-(2,2-dimeth	AR
licorice	(E)-1-(2,4-dihydroxyphenyl)-3-(2,2-dimeth	SCN5A
licorice	(E)-1-(2,4-dihydroxyphenyl)-3-(2,2-dimeth	PPARG
licorice	(E)-1-(2,4-dihydroxyphenyl)-3-(2,2-dimeth	F10
licorice	(E)-1-(2,4-dihydroxyphenyl)-3-(2,2-dimeth	PTGS2
licorice	(E)-1-(2,4-dihydroxyphenyl)-3-(2,2-dimeth	CA2
licorice	(E)-1-(2,4-dihydroxyphenyl)-3-(2,2-dimeth	RXRA
licorice	(E)-1-(2,4-dihydroxyphenyl)-3-(2,2-dimeth	ADRA1B
licorice	(E)-1-(2,4-dihydroxyphenyl)-3-(2,2-dimeth	ESR2
licorice	(E)-1-(2,4-dihydroxyphenyl)-3-(2,2-dimeth	MAPK14
licorice	(E)-1-(2,4-dihydroxyphenyl)-3-(2,2-dimeth	GSK3B
licorice	(E)-1-(2,4-dihydroxyphenyl)-3-(2,2-dimeth	CDK2
licorice	(E)-1-(2,4-dihydroxyphenyl)-3-(2,2-dimeth	CHEK1
licorice	(E)-1-(2,4-dihydroxyphenyl)-3-(2,2-dimeth	PIM1
licorice	(E)-1-(2,4-dihydroxyphenyl)-3-(2,2-dimeth	CCNA2
licorice	(E)-1-(2,4-dihydroxyphenyl)-3-(2,2-dimeth	NCOA2
licorice	(E)-1-(2,4-dihydroxyphenyl)-3-(2,2-dimeth	CALM2

licorice	(2S)-6-(2,4-dihydroxyphenyl)-2-(2-hydroxy	NOS2
licorice	(2S)-6-(2,4-dihydroxyphenyl)-2-(2-hydroxy	F2
licorice	(2S)-6-(2,4-dihydroxyphenyl)-2-(2-hydroxy	ESR1
licorice	(2S)-6-(2,4-dihydroxyphenyl)-2-(2-hydroxy	AR
licorice	(2S)-6-(2,4-dihydroxyphenyl)-2-(2-hydroxy	PPARG
licorice	(2S)-6-(2,4-dihydroxyphenyl)-2-(2-hydroxy	F10
licorice	(2S)-6-(2,4-dihydroxyphenyl)-2-(2-hydroxy	PTGS2
licorice	(2S)-6-(2,4-dihydroxyphenyl)-2-(2-hydroxy	F7
licorice	(2S)-6-(2,4-dihydroxyphenyl)-2-(2-hydroxy	KDR
licorice	(2S)-6-(2,4-dihydroxyphenyl)-2-(2-hydroxy	ACHE
licorice	(2S)-6-(2,4-dihydroxyphenyl)-2-(2-hydroxy	TOP2A
licorice	(2S)-6-(2,4-dihydroxyphenyl)-2-(2-hydroxy	ESR2
licorice	(2S)-6-(2,4-dihydroxyphenyl)-2-(2-hydroxy	DPP4
licorice	(2S)-6-(2,4-dihydroxyphenyl)-2-(2-hydroxy	MAPK14
licorice	(2S)-6-(2,4-dihydroxyphenyl)-2-(2-hydroxy	GSK3B
licorice	(2S)-6-(2,4-dihydroxyphenyl)-2-(2-hydroxy	CDK2
licorice	(2S)-6-(2,4-dihydroxyphenyl)-2-(2-hydroxy	CHEK1
licorice	(2S)-6-(2,4-dihydroxyphenyl)-2-(2-hydroxy	PRSS1
licorice	(2S)-6-(2,4-dihydroxyphenyl)-2-(2-hydroxy	PIM1
licorice	(2S)-6-(2,4-dihydroxyphenyl)-2-(2-hydroxy	CCNA2
licorice	(2S)-6-(2,4-dihydroxyphenyl)-2-(2-hydroxy	CALM2
licorice	Semilicoisoflavone B	NOS2
licorice	Semilicoisoflavone B	F2
licorice	Semilicoisoflavone B	ESR1
licorice	Semilicoisoflavone B	AR
licorice	Semilicoisoflavone B	SCN5A
licorice	Semilicoisoflavone B	PPARG
licorice	Semilicoisoflavone B	F10
licorice	Semilicoisoflavone B	PTGS2
licorice	Semilicoisoflavone B	F7
licorice	Semilicoisoflavone B	ACHE
licorice	Semilicoisoflavone B	TOP2A
licorice	Semilicoisoflavone B	GSK3B
licorice	Semilicoisoflavone B	HSP90AA1
licorice	Semilicoisoflavone B	CDK2
licorice	Semilicoisoflavone B	CHEK1
licorice	Semilicoisoflavone B	PRSS1
licorice	Semilicoisoflavone B	CALM2
licorice	Glepidotin A	NOS2
licorice	Glepidotin A	PTGS1
licorice	Glepidotin A	F2
licorice	Glepidotin A	ESR1
licorice	Glepidotin A	AR
licorice	Glepidotin A	SCN5A
licorice	Glepidotin A	PPARG
licorice	Glepidotin A	F10
licorice	Glepidotin A	PTGS2
licorice	Glepidotin A	NOS3
licorice	Glepidotin A	F7
licorice	Glepidotin A	KDR
licorice	Glepidotin A	RXRA
licorice	Glepidotin A	PDE3A
licorice	Glepidotin A	TOP2A
licorice	Glepidotin A	DPP4
licorice	Glepidotin A	MAPK14
licorice	Glepidotin A	GSK3B
licorice	Glepidotin A	HSP90AA1
licorice	Glepidotin A	CDK2

licorice	Glepidotin A	CHEK1
licorice	Glepidotin A	PRSS1
licorice	Glepidotin A	PIM1
licorice	Glepidotin A	CCNA2
licorice	Glepidotin A	CALM2
licorice	Glepidotin B	PTGS1
licorice	Glepidotin B	ESR1
licorice	Glepidotin B	SCN5A
licorice	Glepidotin B	F10
licorice	Glepidotin B	PTGS2
licorice	Glepidotin B	NOS3
licorice	Glepidotin B	F7
licorice	Glepidotin B	RXRA
licorice	Glepidotin B	PDE3A
licorice	Glepidotin B	ADRA1B
licorice	Glepidotin B	TOP2A
licorice	Glepidotin B	HSP90AA1
licorice	Glepidotin B	NCOA1
licorice	Glepidotin B	CALM2
licorice	Glypallichalcone	NOS2
licorice	Glypallichalcone	PTGS1
licorice	Glypallichalcone	CHRM1
licorice	Glypallichalcone	ESR1
licorice	Glypallichalcone	AR
licorice	Glypallichalcone	SCN5A
licorice	Glypallichalcone	PPARG
licorice	Glypallichalcone	PTGS2
licorice	Glypallichalcone	CA2
licorice	Glypallichalcone	PDE3A
licorice	Glypallichalcone	ADRA1B
licorice	Glypallichalcone	SLC6A3
licorice	Glypallichalcone	ADRB2
licorice	Glypallichalcone	SLC6A4
licorice	Glypallichalcone	ESR2
licorice	Glypallichalcone	MAPK14
licorice	Glypallichalcone	GSK3B
licorice	Glypallichalcone	HSP90AA1
licorice	Glypallichalcone	CDK2
licorice	Glypallichalcone	LTA4H
licorice	Glypallichalcone	MAOB
licorice	Glypallichalcone	CHEK1
licorice	Glypallichalcone	PKIA
licorice	Glypallichalcone	CCNA2
licorice	Glypallichalcone	NCOA1
licorice	Glypallichalcone	PKIA
licorice	Glypallichalcone	CALM2
licorice	8-(6-hydroxy-2-benzofuranyl)-2,2-dimethyl	NOS2
licorice	8-(6-hydroxy-2-benzofuranyl)-2,2-dimethyl	ESR1
licorice	8-(6-hydroxy-2-benzofuranyl)-2,2-dimethyl	PTGS2
licorice	8-(6-hydroxy-2-benzofuranyl)-2,2-dimethyl	RXRA
licorice	8-(6-hydroxy-2-benzofuranyl)-2,2-dimethyl	HSP90AA1
licorice	8-(6-hydroxy-2-benzofuranyl)-2,2-dimethyl	PIK3CG
licorice	Licochalcone B	NOS2
licorice	Licochalcone B	PTGS1
licorice	Licochalcone B	ESR1
licorice	Licochalcone B	AR
licorice	Licochalcone B	PPARG
licorice	Licochalcone B	PTGS2

licorice	Licochalcone B	CA2
licorice	Licochalcone B	PDE3A
licorice	Licochalcone B	ADRB2
licorice	Licochalcone B	ESR2
licorice	Licochalcone B	MAPK14
licorice	Licochalcone B	GSK3B
licorice	Licochalcone B	HSP90AA1
licorice	Licochalcone B	CDK2
licorice	Licochalcone B	CHEK1
licorice	Licochalcone B	PKIA
licorice	Licochalcone B	PIM1
licorice	Licochalcone B	CCNA2
licorice	Licochalcone B	CALM2
licorice	licochalcone G	NOS2
licorice	licochalcone G	ESR1
licorice	licochalcone G	AR
licorice	licochalcone G	PPARG
licorice	licochalcone G	F10
licorice	licochalcone G	PTGS2
licorice	licochalcone G	KDR
licorice	licochalcone G	ESR2
licorice	licochalcone G	MAPK14
licorice	licochalcone G	GSK3B
licorice	licochalcone G	HSP90AA1
licorice	licochalcone G	CDK2
licorice	licochalcone G	PIM1
licorice	licochalcone G	CCNA2
licorice	licochalcone G	NCOA2
licorice	licochalcone G	CALM2
licorice	Licoricone	NOS2
licorice	Licoricone	F2
licorice	Licoricone	KCNH2
licorice	Licoricone	ESR1
licorice	Licoricone	AR
licorice	Licoricone	PPARG
licorice	Licoricone	F10
licorice	Licoricone	PTGS2
licorice	Licoricone	KDR
licorice	Licoricone	TOP2A
licorice	Licoricone	CHEK1
licorice	Licoricone	PRSS1
licorice	Licoricone	PIM1
licorice	Licoricone	NCOA2
licorice	Licoricone	CALM2
licorice	Gancaonin A	NOS2
licorice	Gancaonin A	F2
licorice	Gancaonin A	ESR1
licorice	Gancaonin A	AR
licorice	Gancaonin A	SCN5A
licorice	Gancaonin A	PPARG
licorice	Gancaonin A	F10
licorice	Gancaonin A	PTGS2
licorice	Gancaonin A	ACHE
licorice	Gancaonin A	TOP2A
licorice	Gancaonin A	ESR2
licorice	Gancaonin A	DPP4
licorice	Gancaonin A	GSK3B
licorice	Gancaonin A	HSP90AA1

licorice	Gancaonin A	CHEK1
licorice	Gancaonin A	PRSS1
licorice	Gancaonin A	PIM1
licorice	Gancaonin A	CCNA2
licorice	Gancaonin A	NCOA2
licorice	Gancaonin A	CALM2
licorice	Gancaonin B	NOS2
licorice	Gancaonin B	F2
licorice	Gancaonin B	ESR1
licorice	Gancaonin B	AR
licorice	Gancaonin B	PPARG
licorice	Gancaonin B	F10
licorice	Gancaonin B	PTGS2
licorice	Gancaonin B	F7
licorice	Gancaonin B	KDR
licorice	Gancaonin B	ADRA1B
licorice	Gancaonin B	ADRB2
licorice	Gancaonin B	TOP2A
licorice	Gancaonin B	ESR2
licorice	Gancaonin B	DPP4
licorice	Gancaonin B	GSK3B
licorice	Gancaonin B	HSP90AA1
licorice	Gancaonin B	CHEK1
licorice	Gancaonin B	PRSS1
licorice	Gancaonin B	PIM1
licorice	Gancaonin B	CCNA2
licorice	Gancaonin B	NCOA2
licorice	Gancaonin B	CALM2
licorice	3-(3,4-dihydroxyphenyl)-5,7-dihydroxy-8-(	NOS2
licorice	3-(3,4-dihydroxyphenyl)-5,7-dihydroxy-8-(	F2
licorice	3-(3,4-dihydroxyphenyl)-5,7-dihydroxy-8-(	ESR1
licorice	3-(3,4-dihydroxyphenyl)-5,7-dihydroxy-8-(	AR
licorice	3-(3,4-dihydroxyphenyl)-5,7-dihydroxy-8-(	PPARG
licorice	3-(3,4-dihydroxyphenyl)-5,7-dihydroxy-8-(	F10
licorice	3-(3,4-dihydroxyphenyl)-5,7-dihydroxy-8-(	PTGS2
licorice	3-(3,4-dihydroxyphenyl)-5,7-dihydroxy-8-(	PTPN1
licorice	3-(3,4-dihydroxyphenyl)-5,7-dihydroxy-8-(	MAPK14
licorice	3-(3,4-dihydroxyphenyl)-5,7-dihydroxy-8-(	GSK3B
licorice	3-(3,4-dihydroxyphenyl)-5,7-dihydroxy-8-(	HSP90AA1
licorice	3-(3,4-dihydroxyphenyl)-5,7-dihydroxy-8-(	CDK2
licorice	3-(3,4-dihydroxyphenyl)-5,7-dihydroxy-8-(	CHEK1
licorice	3-(3,4-dihydroxyphenyl)-5,7-dihydroxy-8-(	PRSS1
licorice	3-(3,4-dihydroxyphenyl)-5,7-dihydroxy-8-(	PIM1
licorice	3-(3,4-dihydroxyphenyl)-5,7-dihydroxy-8-(	CCNA2
licorice	3-(3,4-dihydroxyphenyl)-5,7-dihydroxy-8-(	NCOA2
licorice	3-(3,4-dihydroxyphenyl)-5,7-dihydroxy-8-(	CALM2
licorice	5,7-dihydroxy-3-(4-methoxyphenyl)-8-(3-m	NOS2
licorice	5,7-dihydroxy-3-(4-methoxyphenyl)-8-(3-m	KCNH2
licorice	5,7-dihydroxy-3-(4-methoxyphenyl)-8-(3-m	ESR1
licorice	5,7-dihydroxy-3-(4-methoxyphenyl)-8-(3-m	AR
licorice	5,7-dihydroxy-3-(4-methoxyphenyl)-8-(3-m	PPARG
licorice	5,7-dihydroxy-3-(4-methoxyphenyl)-8-(3-m	F10
licorice	5,7-dihydroxy-3-(4-methoxyphenyl)-8-(3-m	PTGS2
licorice	5,7-dihydroxy-3-(4-methoxyphenyl)-8-(3-m	TOP2A
licorice	5,7-dihydroxy-3-(4-methoxyphenyl)-8-(3-m	ESR2
licorice	5,7-dihydroxy-3-(4-methoxyphenyl)-8-(3-m	DPP4
licorice	5,7-dihydroxy-3-(4-methoxyphenyl)-8-(3-m	MAPK14
licorice	5,7-dihydroxy-3-(4-methoxyphenyl)-8-(3-m	GSK3B

licorice	5,7-dihydroxy-3-(4-methoxyphenyl)-8-(3-m	HSP90AA1
licorice	5,7-dihydroxy-3-(4-methoxyphenyl)-8-(3-m	CDK2
licorice	5,7-dihydroxy-3-(4-methoxyphenyl)-8-(3-m	CHEK1
licorice	5,7-dihydroxy-3-(4-methoxyphenyl)-8-(3-m	PRSS1
licorice	5,7-dihydroxy-3-(4-methoxyphenyl)-8-(3-m	PIM1
licorice	5,7-dihydroxy-3-(4-methoxyphenyl)-8-(3-m	CCNA2
licorice	5,7-dihydroxy-3-(4-methoxyphenyl)-8-(3-m	NCOA2
licorice	5,7-dihydroxy-3-(4-methoxyphenyl)-8-(3-m	CALM2
licorice	2-(3,4-dihydroxyphenyl)-5,7-dihydroxy-6-(	F2
licorice	2-(3,4-dihydroxyphenyl)-5,7-dihydroxy-6-(	AR
licorice	2-(3,4-dihydroxyphenyl)-5,7-dihydroxy-6-(	SCN5A
licorice	2-(3,4-dihydroxyphenyl)-5,7-dihydroxy-6-(	PPARG
licorice	2-(3,4-dihydroxyphenyl)-5,7-dihydroxy-6-(	F10
licorice	2-(3,4-dihydroxyphenyl)-5,7-dihydroxy-6-(	PTGS2
licorice	2-(3,4-dihydroxyphenyl)-5,7-dihydroxy-6-(	F7
licorice	2-(3,4-dihydroxyphenyl)-5,7-dihydroxy-6-(	ADRB2
licorice	2-(3,4-dihydroxyphenyl)-5,7-dihydroxy-6-(	DPP4
licorice	2-(3,4-dihydroxyphenyl)-5,7-dihydroxy-6-(	HSP90AA1
licorice	2-(3,4-dihydroxyphenyl)-5,7-dihydroxy-6-(	CDK2
licorice	2-(3,4-dihydroxyphenyl)-5,7-dihydroxy-6-(	CHEK1
licorice	2-(3,4-dihydroxyphenyl)-5,7-dihydroxy-6-(	PRSS1
licorice	2-(3,4-dihydroxyphenyl)-5,7-dihydroxy-6-(	PIM1
licorice	2-(3,4-dihydroxyphenyl)-5,7-dihydroxy-6-(	CCNA2
licorice	2-(3,4-dihydroxyphenyl)-5,7-dihydroxy-6-(	CALM2
licorice	Licocoumarone	ESR1
licorice	Licocoumarone	AR
licorice	Licocoumarone	ESR2
licorice	Licocoumarone	GSK3B
licorice	Licocoumarone	HSP90AA1
licorice	Licocoumarone	CDK2
licorice	Licocoumarone	CCNA2
licorice	Licoisoflavone	NOS2
licorice	Licoisoflavone	F2
licorice	Licoisoflavone	ESR1
licorice	Licoisoflavone	AR
licorice	Licoisoflavone	PPARG
licorice	Licoisoflavone	F10
licorice	Licoisoflavone	PTGS2
licorice	Licoisoflavone	KDR
licorice	Licoisoflavone	TOP2A
licorice	Licoisoflavone	DPP4
licorice	Licoisoflavone	MAPK14
licorice	Licoisoflavone	HSP90AA1
licorice	Licoisoflavone	CDK2
licorice	Licoisoflavone	CHEK1
licorice	Licoisoflavone	PRSS1
licorice	Licoisoflavone	PIM1
licorice	Licoisoflavone	CCNA2
licorice	Licoisoflavone	NCOA2
licorice	Licoisoflavone	CALM2
licorice	Licoisoflavone B	NOS2
licorice	Licoisoflavone B	F2
licorice	Licoisoflavone B	ESR1
licorice	Licoisoflavone B	AR
licorice	Licoisoflavone B	PPARG
licorice	Licoisoflavone B	F10
licorice	Licoisoflavone B	PTGS2
licorice	Licoisoflavone B	ACHE

licorice	Licoisoflavone B	TOP2A
licorice	Licoisoflavone B	ESR2
licorice	Licoisoflavone B	GSK3B
licorice	Licoisoflavone B	CDK2
licorice	Licoisoflavone B	CHEK1
licorice	Licoisoflavone B	PRSS1
licorice	Licoisoflavone B	PIM1
licorice	Licoisoflavone B	CCNA2
licorice	Licoisoflavone B	CALM2
licorice	licoisoflavanone	NOS2
licorice	licoisoflavanone	PTGS1
licorice	licoisoflavanone	ESR1
licorice	licoisoflavanone	AR
licorice	licoisoflavanone	SCN5A
licorice	licoisoflavanone	PPARG
licorice	licoisoflavanone	F10
licorice	licoisoflavanone	PTGS2
licorice	licoisoflavanone	F7
licorice	licoisoflavanone	ACHE
licorice	licoisoflavanone	TOP2A
licorice	licoisoflavanone	ESR2
licorice	licoisoflavanone	GSK3B
licorice	licoisoflavanone	HSP90AA1
licorice	licoisoflavanone	CDK2
licorice	licoisoflavanone	PRSS1
licorice	licoisoflavanone	PIM1
licorice	licoisoflavanone	CCNA2
licorice	licoisoflavanone	NCOA1
licorice	licoisoflavanone	CALM2
licorice	shinpterocarpin	NOS2
licorice	shinpterocarpin	PTGS1
licorice	shinpterocarpin	CHRM3
licorice	shinpterocarpin	KCNH2
licorice	shinpterocarpin	CHRM1
licorice	shinpterocarpin	ESR1
licorice	shinpterocarpin	AR
licorice	shinpterocarpin	SCN5A
licorice	shinpterocarpin	PPARG
licorice	shinpterocarpin	PTGS2
licorice	shinpterocarpin	RXRA
licorice	shinpterocarpin	OPRD1
licorice	shinpterocarpin	ADRA1B
licorice	shinpterocarpin	ADRB2
licorice	shinpterocarpin	ADRA1D
licorice	shinpterocarpin	OPRM1
licorice	shinpterocarpin	ESR2
licorice	shinpterocarpin	MAPK14
licorice	shinpterocarpin	GSK3B
licorice	shinpterocarpin	CDK2
licorice	shinpterocarpin	PIK3CG
licorice	shinpterocarpin	CHRNA7
licorice	shinpterocarpin	PKIA
licorice	shinpterocarpin	PRSS1
licorice	shinpterocarpin	PIM1
licorice	shinpterocarpin	CCNA2
licorice	shinpterocarpin	NCOA1
licorice	shinpterocarpin	CALM2
licorice	(E)-3-[3,4-dihydroxy-5-(3-methylbut-2-eny	ESR1

licorice	(E)-3-[3,4-dihydroxy-5-(3-methylbut-2-eny	AR
licorice	(E)-3-[3,4-dihydroxy-5-(3-methylbut-2-eny	PPARG
licorice	(E)-3-[3,4-dihydroxy-5-(3-methylbut-2-eny	PTGS2
licorice	(E)-3-[3,4-dihydroxy-5-(3-methylbut-2-eny	MAPK14
licorice	(E)-3-[3,4-dihydroxy-5-(3-methylbut-2-eny	GSK3B
licorice	(E)-3-[3,4-dihydroxy-5-(3-methylbut-2-eny	HSP90AA1
licorice	(E)-3-[3,4-dihydroxy-5-(3-methylbut-2-eny	CDK2
licorice	(E)-3-[3,4-dihydroxy-5-(3-methylbut-2-eny	PIM1
licorice	(E)-3-[3,4-dihydroxy-5-(3-methylbut-2-eny	CCNA2
licorice	(E)-3-[3,4-dihydroxy-5-(3-methylbut-2-eny	NCOA2
licorice	(E)-3-[3,4-dihydroxy-5-(3-methylbut-2-eny	CALM2
licorice	Glyzaglabrin	NOS2
licorice	Glyzaglabrin	PTGS1
licorice	Glyzaglabrin	ESR1
licorice	Glyzaglabrin	AR
licorice	Glyzaglabrin	PPARG
licorice	Glyzaglabrin	PTGS2
licorice	Glyzaglabrin	ESR2
licorice	Glyzaglabrin	DPP4
licorice	Glyzaglabrin	MAPK14
licorice	Glyzaglabrin	GSK3B
licorice	Glyzaglabrin	HSP90AA1
licorice	Glyzaglabrin	CDK2
licorice	Glyzaglabrin	PIK3CG
licorice	Glyzaglabrin	CHEK1
licorice	Glyzaglabrin	PKIA
licorice	Glyzaglabrin	PRSS1
licorice	Glyzaglabrin	PIM1
licorice	Glyzaglabrin	CCNA2
licorice	Glabranin	NOS2
licorice	Glabranin	PTGS1
licorice	Glabranin	ESR1
licorice	Glabranin	SCN5A
licorice	Glabranin	F10
licorice	Glabranin	PTGS2
licorice	Glabranin	NOS3
licorice	Glabranin	PDE3A
licorice	Glabranin	HSP90AA1
licorice	Glabranin	PKIA
licorice	Glabranin	CALM2
licorice	Glabrone	NOS2
licorice	Glabrone	PTGS1
licorice	Glabrone	F2
licorice	Glabrone	ESR1
licorice	Glabrone	AR
licorice	Glabrone	SCN5A
licorice	Glabrone	PPARG
licorice	Glabrone	F10
licorice	Glabrone	PTGS2
licorice	Glabrone	RXRA
licorice	Glabrone	ACHE
licorice	Glabrone	ESR2
licorice	Glabrone	DPP4
licorice	Glabrone	MAPK14
licorice	Glabrone	GSK3B
licorice	Glabrone	CDK2
licorice	Glabrone	CHEK1
licorice	Glabrone	PRSS1

licorice	Glabrone	PIM1
licorice	Glabrone	CCNA2
licorice	Glabrone	CALM2
licorice	1,3-dihydroxy-9-methoxy-6-benzofurano[3,	ESR1
licorice	1,3-dihydroxy-9-methoxy-6-benzofurano[3,	PPARG
licorice	1,3-dihydroxy-9-methoxy-6-benzofurano[3,	ESR2
licorice	1,3-dihydroxy-9-methoxy-6-benzofurano[3,	MAPK14
licorice	1,3-dihydroxy-9-methoxy-6-benzofurano[3,	GSK3B
licorice	1,3-dihydroxy-9-methoxy-6-benzofurano[3,	HSP90AA1
licorice	1,3-dihydroxy-9-methoxy-6-benzofurano[3,	CDK2
licorice	1,3-dihydroxy-9-methoxy-6-benzofurano[3,	CHEK1
licorice	1,3-dihydroxy-9-methoxy-6-benzofurano[3,	PKIA
licorice	1,3-dihydroxy-9-methoxy-6-benzofurano[3,	CCNA2
licorice	1,3-dihydroxy-8,9-dimethoxy-6-benzofuran	ESR1
licorice	1,3-dihydroxy-8,9-dimethoxy-6-benzofuran	AR
licorice	1,3-dihydroxy-8,9-dimethoxy-6-benzofuran	PPARG
licorice	1,3-dihydroxy-8,9-dimethoxy-6-benzofuran	MAPK14
licorice	1,3-dihydroxy-8,9-dimethoxy-6-benzofuran	GSK3B
licorice	1,3-dihydroxy-8,9-dimethoxy-6-benzofuran	HSP90AA1
licorice	1,3-dihydroxy-8,9-dimethoxy-6-benzofuran	CDK2
licorice	1,3-dihydroxy-8,9-dimethoxy-6-benzofuran	CHEK1
licorice	1,3-dihydroxy-8,9-dimethoxy-6-benzofuran	PKIA
licorice	Eurycarpin A	NOS2
licorice	Eurycarpin A	F2
licorice	Eurycarpin A	ESR1
licorice	Eurycarpin A	AR
licorice	Eurycarpin A	SCN5A
licorice	Eurycarpin A	PPARG
licorice	Eurycarpin A	F10
licorice	Eurycarpin A	PTGS2
licorice	Eurycarpin A	ESR2
licorice	Eurycarpin A	DPP4
licorice	Eurycarpin A	MAPK14
licorice	Eurycarpin A	GSK3B
licorice	Eurycarpin A	HSP90AA1
licorice	Eurycarpin A	CDK2
licorice	Eurycarpin A	CHEK1
licorice	Eurycarpin A	PRSS1
licorice	Eurycarpin A	PIM1
licorice	Eurycarpin A	CCNA2
licorice	Eurycarpin A	CALM2
licorice	Sigmoidin-B	ESR1
licorice	Sigmoidin-B	F10
licorice	Sigmoidin-B	PTGS2
licorice	Sigmoidin-B	KDR
licorice	Sigmoidin-B	HSP90AA1
licorice	Sigmoidin-B	CALM2
licorice	(2R)-7-hydroxy-2-(4-hydroxyphenyl)chrom	PTGS1
licorice	(2R)-7-hydroxy-2-(4-hydroxyphenyl)chrom	ESR1
licorice	(2R)-7-hydroxy-2-(4-hydroxyphenyl)chrom	PTGS2
licorice	(2R)-7-hydroxy-2-(4-hydroxyphenyl)chrom	RXRA
licorice	(2R)-7-hydroxy-2-(4-hydroxyphenyl)chrom	PDE3A
licorice	(2R)-7-hydroxy-2-(4-hydroxyphenyl)chrom	ADRB2
licorice	(2R)-7-hydroxy-2-(4-hydroxyphenyl)chrom	HSP90AA1
licorice	(2R)-7-hydroxy-2-(4-hydroxyphenyl)chrom	PIK3CG
licorice	(2R)-7-hydroxy-2-(4-hydroxyphenyl)chrom	LACTBL1
licorice	(2R)-7-hydroxy-2-(4-hydroxyphenyl)chrom	MAOB
licorice	(2R)-7-hydroxy-2-(4-hydroxyphenyl)chrom	PKIA

licorice	(2R)-7-hydroxy-2-(4-hydroxyphenyl)chrom	PKIA
licorice	(2R)-7-hydroxy-2-(4-hydroxyphenyl)chrom	CALM2
licorice	(2R)-7-hydroxy-2-(4-hydroxyphenyl)chrom	GABRA1
licorice	(2R)-7-hydroxy-2-(4-hydroxyphenyl)chrom	SLC6A4
licorice	(2S)-7-hydroxy-2-(4-hydroxyphenyl)-8-(3-r	NOS2
licorice	(2S)-7-hydroxy-2-(4-hydroxyphenyl)-8-(3-r	PTGS1
licorice	(2S)-7-hydroxy-2-(4-hydroxyphenyl)-8-(3-r	ESR1
licorice	(2S)-7-hydroxy-2-(4-hydroxyphenyl)-8-(3-r	SCN5A
licorice	(2S)-7-hydroxy-2-(4-hydroxyphenyl)-8-(3-r	F10
licorice	(2S)-7-hydroxy-2-(4-hydroxyphenyl)-8-(3-r	PTGS2
licorice	(2S)-7-hydroxy-2-(4-hydroxyphenyl)-8-(3-r	PDE3A
licorice	(2S)-7-hydroxy-2-(4-hydroxyphenyl)-8-(3-r	ADRA1B
licorice	(2S)-7-hydroxy-2-(4-hydroxyphenyl)-8-(3-r	ADRB2
licorice	(2S)-7-hydroxy-2-(4-hydroxyphenyl)-8-(3-r	ESR2
licorice	(2S)-7-hydroxy-2-(4-hydroxyphenyl)-8-(3-r	HSP90AA1
licorice	(2S)-7-hydroxy-2-(4-hydroxyphenyl)-8-(3-r	CALM2
licorice	Isoglycyrol	NOS2
licorice	Isoglycyrol	ESR1
licorice	Isoglycyrol	AR
licorice	Isoglycyrol	PTGS2
licorice	Isoglycyrol	DPP4
licorice	Isoglycyrol	GSK3B
licorice	Isoglycyrol	PIM1
licorice	Isolicoflavonol	NOS2
licorice	Isolicoflavonol	F2
licorice	Isolicoflavonol	ESR1
licorice	Isolicoflavonol	AR
licorice	Isolicoflavonol	PPARG
licorice	Isolicoflavonol	F10
licorice	Isolicoflavonol	PTGS2
licorice	Isolicoflavonol	GSK3B
licorice	Isolicoflavonol	HSP90AA1
licorice	Isolicoflavonol	CDK2
licorice	Isolicoflavonol	PRSS1
licorice	Isolicoflavonol	PIM1
licorice	Isolicoflavonol	CCNA2
licorice	Isolicoflavonol	NCOA2
licorice	Isolicoflavonol	CALM2
licorice	HMO	NOS2
licorice	HMO	PTGS1
licorice	HMO	CHRM1
licorice	HMO	ESR1
licorice	HMO	AR
licorice	HMO	SCN5A
licorice	HMO	PPARG
licorice	HMO	PTGS2
licorice	HMO	RXRA
licorice	HMO	PDE3A
licorice	HMO	SLC6A3
licorice	HMO	ADRB2
licorice	HMO	SLC6A4
licorice	HMO	ESR2
licorice	HMO	DPP4
licorice	HMO	MAPK14
licorice	HMO	GSK3B
licorice	HMO	CDK2
licorice	HMO	MAOB
licorice	HMO	CHEK1

licorice	HMO	PKIA
licorice	HMO	PRSS1
licorice	HMO	PIM1
licorice	HMO	CCNA2
licorice	HMO	PKIA
licorice	HMO	CALM2
licorice	1-Methoxyphaseollidin	NOS2
licorice	1-Methoxyphaseollidin	PTGS1
licorice	1-Methoxyphaseollidin	F2
licorice	1-Methoxyphaseollidin	KCNH2
licorice	1-Methoxyphaseollidin	ESR1
licorice	1-Methoxyphaseollidin	AR
licorice	1-Methoxyphaseollidin	SCN5A
licorice	1-Methoxyphaseollidin	PPARG
licorice	1-Methoxyphaseollidin	F10
licorice	1-Methoxyphaseollidin	PTGS2
licorice	1-Methoxyphaseollidin	NOS3
licorice	1-Methoxyphaseollidin	KDR
licorice	1-Methoxyphaseollidin	RXRA
licorice	1-Methoxyphaseollidin	ADRA1B
licorice	1-Methoxyphaseollidin	ADRB2
licorice	1-Methoxyphaseollidin	ADRA1D
licorice	1-Methoxyphaseollidin	TOP2A
licorice	1-Methoxyphaseollidin	ESR2
licorice	1-Methoxyphaseollidin	MAPK14
licorice	1-Methoxyphaseollidin	GSK3B
licorice	1-Methoxyphaseollidin	HSP90AA1
licorice	1-Methoxyphaseollidin	CDK2
licorice	1-Methoxyphaseollidin	PIK3CG
licorice	1-Methoxyphaseollidin	PRSS1
licorice	1-Methoxyphaseollidin	PIM1
licorice	1-Methoxyphaseollidin	CCNA2
licorice	1-Methoxyphaseollidin	NCOA2
licorice	1-Methoxyphaseollidin	NCOA1
licorice	1-Methoxyphaseollidin	CALM2
licorice	Quercetin der.	NOS2
licorice	Quercetin der.	PTGS1
licorice	Quercetin der.	ESR1
licorice	Quercetin der.	AR
licorice	Quercetin der.	SCN5A
licorice	Quercetin der.	PPARG
licorice	Quercetin der.	PTGS2
licorice	Quercetin der.	PTPN1
licorice	Quercetin der.	ESR2
licorice	Quercetin der.	DPP4
licorice	Quercetin der.	MAPK14
licorice	Quercetin der.	GSK3B
licorice	Quercetin der.	HSP90AA1
licorice	Quercetin der.	CDK2
licorice	Quercetin der.	PRSS1
licorice	Quercetin der.	NCOA2
licorice	Quercetin der.	CALM2
licorice	6-prenylated eriodictyol	NOS2
licorice	6-prenylated eriodictyol	ESR1
licorice	6-prenylated eriodictyol	SCN5A
licorice	6-prenylated eriodictyol	F10
licorice	6-prenylated eriodictyol	PTGS2
licorice	6-prenylated eriodictyol	F7

licorice	6-prenylated eriodictyol	HSP90AA1
licorice	6-prenylated eriodictyol	CALM2
licorice	7-Acetoxy-2-methylisoflavone	NOS2
licorice	7-Acetoxy-2-methylisoflavone	PTGS1
licorice	7-Acetoxy-2-methylisoflavone	F2
licorice	7-Acetoxy-2-methylisoflavone	ESR1
licorice	7-Acetoxy-2-methylisoflavone	AR
licorice	7-Acetoxy-2-methylisoflavone	SCN5A
licorice	7-Acetoxy-2-methylisoflavone	PPARG
licorice	7-Acetoxy-2-methylisoflavone	PTGS2
licorice	7-Acetoxy-2-methylisoflavone	NOS3
licorice	7-Acetoxy-2-methylisoflavone	RXRA
licorice	7-Acetoxy-2-methylisoflavone	ACHE
licorice	7-Acetoxy-2-methylisoflavone	PDE3A
licorice	7-Acetoxy-2-methylisoflavone	ADRA1B
licorice	7-Acetoxy-2-methylisoflavone	ADRB2
licorice	7-Acetoxy-2-methylisoflavone	ADRA1D
licorice	7-Acetoxy-2-methylisoflavone	GABRA1
licorice	7-Acetoxy-2-methylisoflavone	DPP4
licorice	7-Acetoxy-2-methylisoflavone	MAPK14
licorice	7-Acetoxy-2-methylisoflavone	GSK3B
licorice	7-Acetoxy-2-methylisoflavone	HSP90AA1
licorice	7-Acetoxy-2-methylisoflavone	CDK2
licorice	7-Acetoxy-2-methylisoflavone	CHEK1
licorice	7-Acetoxy-2-methylisoflavone	PRSS1
licorice	7-Acetoxy-2-methylisoflavone	NCOA2
licorice	7-Acetoxy-2-methylisoflavone	CALM2
licorice	8-prenylated eriodictyol	ESR1
licorice	8-prenylated eriodictyol	SCN5A
licorice	8-prenylated eriodictyol	F10
licorice	8-prenylated eriodictyol	PTGS2
licorice	8-prenylated eriodictyol	F7
licorice	8-prenylated eriodictyol	HSP90AA1
licorice	8-prenylated eriodictyol	NCOA1
licorice	8-prenylated eriodictyol	CALM2
licorice	Gancaonin G	NOS2
licorice	Gancaonin G	F2
licorice	Gancaonin G	ESR1
licorice	Gancaonin G	AR
licorice	Gancaonin G	PPARG
licorice	Gancaonin G	F10
licorice	Gancaonin G	PTGS2
licorice	Gancaonin G	NOS3
licorice	Gancaonin G	TOP2A
licorice	Gancaonin G	ESR2
licorice	Gancaonin G	DPP4
licorice	Gancaonin G	MAPK14
licorice	Gancaonin G	GSK3B
licorice	Gancaonin G	HSP90AA1
licorice	Gancaonin G	CHEK1
licorice	Gancaonin G	PRSS1
licorice	Gancaonin G	PIM1
licorice	Gancaonin G	CCNA2
licorice	Gancaonin G	NCOA2
licorice	Gancaonin G	CALM2
licorice	Gancaonin H	ESR1
licorice	Gancaonin H	AR
licorice	Gancaonin H	F10

licorice	Gancaonin H	PTGS2
licorice	Gancaonin H	KDR
licorice	Gancaonin H	TOP2A
licorice	Gancaonin H	HSP90AA1
licorice	Gancaonin H	PRSS1
licorice	Gancaonin H	PIM1
licorice	Gancaonin H	CCNA2
licorice	Gancaonin H	NCOA2
licorice	Gancaonin H	CALM2
licorice	Licoagrocarpin	NOS2
licorice	Licoagrocarpin	PTGS1
licorice	Licoagrocarpin	CHRM3
licorice	Licoagrocarpin	F2
licorice	Licoagrocarpin	KCNH2
licorice	Licoagrocarpin	CHRM1
licorice	Licoagrocarpin	ESR1
licorice	Licoagrocarpin	AR
licorice	Licoagrocarpin	SCN5A
licorice	Licoagrocarpin	PPARG
licorice	Licoagrocarpin	F10
licorice	Licoagrocarpin	CHRM5
licorice	Licoagrocarpin	PTGS2
licorice	Licoagrocarpin	NOS3
licorice	Licoagrocarpin	RXRA
licorice	Licoagrocarpin	ACHE
licorice	Licoagrocarpin	ADRA1B
licorice	Licoagrocarpin	ADRB2
licorice	Licoagrocarpin	ESR2
licorice	Licoagrocarpin	MAPK14
licorice	Licoagrocarpin	GSK3B
licorice	Licoagrocarpin	HSP90AA1
licorice	Licoagrocarpin	CDK2
licorice	Licoagrocarpin	PRSS1
licorice	Licoagrocarpin	PIM1
licorice	Licoagrocarpin	CCNA2
licorice	Licoagrocarpin	NCOA2
licorice	Licoagrocarpin	CALM2
licorice	Glyasperins M	NOS2
licorice	Glyasperins M	PTGS1
licorice	Glyasperins M	KCNH2
licorice	Glyasperins M	ESR1
licorice	Glyasperins M	AR
licorice	Glyasperins M	SCN5A
licorice	Glyasperins M	PPARG
licorice	Glyasperins M	F10
licorice	Glyasperins M	PTGS2
licorice	Glyasperins M	F7
licorice	Glyasperins M	KDR
licorice	Glyasperins M	ACHE
licorice	Glyasperins M	TOP2A
licorice	Glyasperins M	ESR2
licorice	Glyasperins M	PPARD
licorice	Glyasperins M	GSK3B
licorice	Glyasperins M	HSP90AA1
licorice	Glyasperins M	CDK2
licorice	Glyasperins M	PKIA
licorice	Glyasperins M	PRSS1
licorice	Glyasperins M	PIM1

licorice	Glyasperins M	CCNA2
licorice	Glyasperins M	NCOA2
licorice	Glyasperins M	NCOA1
licorice	Glyasperins M	KCNMA1
licorice	Glyasperins M	CALM2
licorice	Licoagroisoflavone	NOS2
licorice	Licoagroisoflavone	F2
licorice	Licoagroisoflavone	ESR1
licorice	Licoagroisoflavone	AR
licorice	Licoagroisoflavone	SCN5A
licorice	Licoagroisoflavone	PPARG
licorice	Licoagroisoflavone	F10
licorice	Licoagroisoflavone	PTGS2
licorice	Licoagroisoflavone	ESR2
licorice	Licoagroisoflavone	DPP4
licorice	Licoagroisoflavone	MAPK14
licorice	Licoagroisoflavone	GSK3B
licorice	Licoagroisoflavone	CDK2
licorice	Licoagroisoflavone	CHEK1
licorice	Licoagroisoflavone	PRSS1
licorice	Licoagroisoflavone	PIM1
licorice	Licoagroisoflavone	CCNA2
licorice	Licoagroisoflavone	CALM2
licorice	Odoratin	NOS2
licorice	Odoratin	PTGS1
licorice	Odoratin	ESR1
licorice	Odoratin	AR
licorice	Odoratin	SCN5A
licorice	Odoratin	PPARG
licorice	Odoratin	PTGS2
licorice	Odoratin	RXRA
licorice	Odoratin	ESR2
licorice	Odoratin	DPP4
licorice	Odoratin	MAPK14
licorice	Odoratin	GSK3B
licorice	Odoratin	HSP90AA1
licorice	Odoratin	CDK2
licorice	Odoratin	CHEK1
licorice	Odoratin	PRSS1
licorice	Odoratin	PIM1
licorice	Odoratin	CCNA2
licorice	Odoratin	NCOA2
licorice	Odoratin	CALM2
licorice	Phaseol	F2
licorice	Phaseol	ESR1
licorice	Phaseol	AR
licorice	Phaseol	PPARG
licorice	Phaseol	PTGS2
licorice	Phaseol	KDR
licorice	Phaseol	MAPK14
licorice	Phaseol	GSK3B
licorice	Phaseol	HSP90AA1
licorice	Phaseol	CDK2
licorice	Phaseol	CHEK1
licorice	Phaseol	PKIA
licorice	Phaseol	PIM1
licorice	Phaseol	CCNA2
licorice	Xambioona	NOS2

licorice	Xambioona	ESR1
licorice	Xambioona	F10
licorice	Xambioona	PTGS2
licorice	Xambioona	ESR2
licorice	Xambioona	PIM1
licorice	Xambioona	NCOA2
licorice	Xambioona	CALM2
Chimonanthus salicifolius	luteolin-5-O-glucoside	TNF
Chimonanthus salicifolius	luteolin-5-O-glucoside	IL2
Chimonanthus salicifolius	Quercetin	NOX4
Chimonanthus salicifolius	Quercetin	AVPR2
Chimonanthus salicifolius	Quercetin	AKR1B1
Chimonanthus salicifolius	Quercetin	XDH
Chimonanthus salicifolius	Quercetin	MAOA
Chimonanthus salicifolius	Quercetin	IGF1R
Chimonanthus salicifolius	Quercetin	FLT3
Chimonanthus salicifolius	Quercetin	CYP19A1
Chimonanthus salicifolius	Quercetin	EGFR
Chimonanthus salicifolius	Quercetin	F2
Chimonanthus salicifolius	Quercetin	CA2
Chimonanthus salicifolius	Quercetin	PIM1
Chimonanthus salicifolius	Quercetin	ALOX5
Chimonanthus salicifolius	Quercetin	AURKB
Chimonanthus salicifolius	Quercetin	DRD4
Chimonanthus salicifolius	Quercetin	ADORA1
Chimonanthus salicifolius	Quercetin	CA7
Chimonanthus salicifolius	Quercetin	GLO1
Chimonanthus salicifolius	Quercetin	MPO
Chimonanthus salicifolius	Quercetin	PIK3R1
Chimonanthus salicifolius	Quercetin	ADORA2A
Chimonanthus salicifolius	Quercetin	DAPK1
Chimonanthus salicifolius	Quercetin	PYGL
Chimonanthus salicifolius	Quercetin	CA1
Chimonanthus salicifolius	Quercetin	GSK3B
Chimonanthus salicifolius	Quercetin	SRC
Chimonanthus salicifolius	Quercetin	PTK2
Chimonanthus salicifolius	Quercetin	HSD17B2
Chimonanthus salicifolius	Quercetin	KDR
Chimonanthus salicifolius	Quercetin	MMP13
Chimonanthus salicifolius	kaempferol	NOS2
Chimonanthus salicifolius	kaempferol	PTGS1
Chimonanthus salicifolius	kaempferol	AR
Chimonanthus salicifolius	kaempferol	PPARG
Chimonanthus salicifolius	kaempferol	PTGS2
Chimonanthus salicifolius	kaempferol	HSP90AA1
Chimonanthus salicifolius	kaempferol	PIK3CG
Chimonanthus salicifolius	kaempferol	PKIA
Chimonanthus salicifolius	kaempferol	NCOA2
Chimonanthus salicifolius	kaempferol	DPP4
Chimonanthus salicifolius	kaempferol	PRSS1
Chimonanthus salicifolius	kaempferol	PGR
Chimonanthus salicifolius	kaempferol	F2
Chimonanthus salicifolius	kaempferol	CHRM1
Chimonanthus salicifolius	kaempferol	NOS3
Chimonanthus salicifolius	kaempferol	GABRA2
Chimonanthus salicifolius	kaempferol	ACHE
Chimonanthus salicifolius	kaempferol	SLC6A2
Chimonanthus salicifolius	kaempferol	CHRM2

Chimonanthus salicifolius	kaempferol	ADRA1B
Chimonanthus salicifolius	kaempferol	GABRA1
Chimonanthus salicifolius	kaempferol	TOP2A
Chimonanthus salicifolius	kaempferol	F7
Chimonanthus salicifolius	kaempferol	CALM2
Chimonanthus salicifolius	kaempferol	RELA
Chimonanthus salicifolius	kaempferol	IKBKB
Chimonanthus salicifolius	kaempferol	AKT1
Chimonanthus salicifolius	kaempferol	BCL2
Chimonanthus salicifolius	kaempferol	BAX
Chimonanthus salicifolius	kaempferol	CD40LG
Chimonanthus salicifolius	kaempferol	JUN
Chimonanthus salicifolius	kaempferol	AHSA1
Chimonanthus salicifolius	kaempferol	CASP3
Chimonanthus salicifolius	kaempferol	MAPK8
Chimonanthus salicifolius	kaempferol	XDH
Chimonanthus salicifolius	kaempferol	MMP1
Chimonanthus salicifolius	kaempferol	STAT1
Chimonanthus salicifolius	kaempferol	CDK1
Chimonanthus salicifolius	kaempferol	PPARG
Chimonanthus salicifolius	kaempferol	HMOX1
Chimonanthus salicifolius	kaempferol	CYP3A4
Chimonanthus salicifolius	kaempferol	CYP1A1
Chimonanthus salicifolius	kaempferol	ICAM1
Chimonanthus salicifolius	kaempferol	SELE
Chimonanthus salicifolius	kaempferol	VCAM1
Chimonanthus salicifolius	kaempferol	NR1I2
Chimonanthus salicifolius	kaempferol	CYP1B1
Chimonanthus salicifolius	kaempferol	ALOX5
Chimonanthus salicifolius	kaempferol	HAS2
Chimonanthus salicifolius	kaempferol	AHR
Chimonanthus salicifolius	kaempferol	PSMD3
Chimonanthus salicifolius	kaempferol	SLC2A4
Chimonanthus salicifolius	kaempferol	NR1I3
Chimonanthus salicifolius	kaempferol	INSR
Chimonanthus salicifolius	kaempferol	DIO1
Chimonanthus salicifolius	kaempferol	GSTM1
Chimonanthus salicifolius	kaempferol	GSTM2
Chimonanthus salicifolius	kaempferol	AKR1C3
Chimonanthus salicifolius	kaempferol	SLPI
