# Supplementary material for: Network Pharmacology-Based Study on the Active Ingredients and Mechanism of Pan Ji Sheng Traditional Chinese Medicine Formula in the Treatment of Inflammation
Source: Evid Based Complement Alternat Med. 2022 Sep 28;2022:5340933. doi: 10.1155/2022/5340933 (PMC9534616; doi:10.1155/2022/5340933)
Supplement: Supplementary Materials — Table S1: targets of Pan Ji Sheng Formula. Table S2: detailed information of herbal-key bioactive ingredients-top 25 hub targets. [file 5340933.f1.zip › Table S2.pdf]

**Herbal Medicine**	**Key Bioactive Ingredients**	**Hub Targets**
Microctis Folium	kaempferol	CASP3
Polygonum chinense	kaempferol	CASP3
Polygonum chinense	luteolin	CASP3
Ecliptae Herba	acacetin	CASP3
Ecliptae Herba	luteolin	CASP3
Perilla Frutescens	luteolin	CASP3
Isatidis Radix	acacetin	CASP3
Chrysanthemi Flos	acacetin	CASP3
Chrysanthemi Flos	kaempferol	CASP3
Chrysanthemi Flos	luteolin	CASP3
Chrysanthemi Flos	naringenin	CASP3
Licorice	kaempferol	CASP3
Licorice	naringenin	CASP3
Chimonanthus salicifolius	kaempferol	CASP3
Microctis Folium	kaempferol	AKT1
Polygonum chinense	kaempferol	AKT1
Polygonum chinense	3-O-methylellagic acid	AKT1
Polygonum chinense	3,3'-Di-O-Methylellagic Acid	AKT1
Polygonum chinense	luteolin	AKT1
Ecliptae Herba	luteolin	AKT1
Perilla Frutescens	luteolin	AKT1
Chrysanthemi Flos	kaempferol	AKT1
Chrysanthemi Flos	luteolin	AKT1
Chrysanthemi Flos	naringenin	AKT1
Licorice	kaempferol	AKT1
Licorice	naringenin	AKT1
Chimonanthus salicifolius	kaempferol	AKT1
Microctis Folium	kaempferol	JUN
Polygonum chinense	kaempferol	JUN
Polygonum chinense	luteolin	JUN
Ecliptae Herba	luteolin	JUN
Perilla Frutescens	luteolin	JUN
Chrysanthemi Flos	kaempferol	JUN
Chrysanthemi Flos	luteolin	JUN
Licorice	formononetin	JUN
Licorice	kaempferol	JUN
Chimonanthus salicifolius	kaempferol	JUN
Licorice	licochalcone a	STAT3
Polygonum chinense	luteolin	TP53
Ecliptae Herba	acacetin	TP53
Ecliptae Herba	luteolin	TP53
Perilla Frutescens	luteolin	TP53
Isatidis Radix	acacetin	TP53
Chrysanthemi Flos	acacetin	TP53
Chrysanthemi Flos	luteolin	TP53
Polygonum chinense	luteolin	MMP9
Ecliptae Herba	luteolin	MMP9
Perilla Frutescens	luteolin	MMP9
Chrysanthemi Flos	luteolin	MMP9
Polygonum chinense	luteolin	BCL2L1
Ecliptae Herba	luteolin	BCL2L1
Perilla Frutescens	luteolin	BCL2L1
Chrysanthemi Flos	luteolin	BCL2L1
Polygonum chinense	Quercetin	SRC
Polygonum chinense	3-O-methylellagic acid	SRC
Polygonum chinense	3,3'-Di-O-Methylellagic Acid	SRC
Chimonanthus salicifolius	Quercetin	SRC

Ecliptae Herba	acacetin	CASP8
Isatidis Radix	acacetin	CASP8
Chrysanthemi Flos	acacetin	CASP8
Polygonum chinense	3-O-methylellagic acid	CCND1
Polygonum chinense	3,3'-Di-O-Methylellagic Acid	CCND1
Polygonum chinense	luteolin	CCND1
Ecliptae Herba	luteolin	CCND1
Perilla Frutescens	luteolin	CCND1
Chrysanthemi Flos	luteolin	CCND1
Licorice	licochalcone a	CCND1
Microctis Folium	isorhamnetin	HSP90AA1
Microctis Folium	kaempferol	HSP90AA1
Microctis Folium	4',5-Dihydroxyflavone	HSP90AA1
Polygonum chinense	kaempferol	HSP90AA1
Polygonum chinense	isorhamnetin	HSP90AA1
Polygonum chinense	luteolin	HSP90AA1
Ecliptae Herba	acacetin	HSP90AA1
Ecliptae Herba	butin	HSP90AA1
Ecliptae Herba	1,3,8,9-tetrahydroxybenzofurano[3,2-c]chromen-6-one	HSP90AA1
Ecliptae Herba	3'-O-Methylorobol	HSP90AA1
Ecliptae Herba	Pratensein	HSP90AA1
Ecliptae Herba	wedelolactone	HSP90AA1
Ecliptae Herba	luteolin	HSP90AA1
Perilla Frutescens	luteolin	HSP90AA1
Isatidis Radix	acacetin	HSP90AA1
Isatidis Radix	EUPATORIN	HSP90AA1
Isatidis Radix	Dinatin	HSP90AA1
Isatidis Radix	hydroxyindirubin	HSP90AA1
Isatidis Radix	(2Z)-2-(2-oxoindolin-3-ylidene)indolin-3-one	HSP90AA1
Isatidis Radix	2-(9-((3-methyl-2-oxopent-3-en-1-yl)oxy)-2-oxo-1,2,8,9-tetrahydrofuro[2,3-h]quinolin-8-yl)propan-2-yl acetate	HSP90AA1
Isatidis Radix	DFV	HSP90AA1
Isatidis Radix	(E)-2-[(3-indole)cyanomethylene-]-3-[illegible]	HSP90AA1
Isatidis Radix	neohesperidin_qt	HSP90AA1
Isatidis Radix	Sinensetin	HSP90AA1
Isatidis Radix	(E)-3-(3,5-dimethoxy-4-hydroxy-benzylidene)-2-indolinone	HSP90AA1
Isatidis Radix	(E)-3-(3,5-dimethoxy-4-hydroxyb-enzylidene)-2-indolinone	HSP90AA1
Isatidis Radix	3-[(3,5-dimethoxy-4-oxo-1-cyclohexa-2,5-dienylidene)methyl]-2,4-dihydro-1H-pyrrolo[2,1-b]quinazolin-9-one	HSP90AA1
Chrysanthemi Flos	acacetin	HSP90AA1
Chrysanthemi Flos	Chryseriol	HSP90AA1
Chrysanthemi Flos	isorhamnetin	HSP90AA1
Chrysanthemi Flos	kaempferol	HSP90AA1
Chrysanthemi Flos	5,7-dihydroxy-2-(3-hydroxy-4-methoxyphenyl)chroman-4-one	HSP90AA1
Chrysanthemi Flos	luteolin	HSP90AA1
Chrysanthemi Flos	EUPATORIN	HSP90AA1
Chrysanthemi Flos	Diosmetin	HSP90AA1
Chrysanthemi Flos	naringenin	HSP90AA1
Chrysanthemi Flos	Artemetin	HSP90AA1

Licorice	Jaranol	HSP90AA1
Licorice	isorhamnetin	HSP90AA1
Licorice	formononetin	HSP90AA1
Licorice	Calycosin	HSP90AA1
Licorice	kaempferol	HSP90AA1
Licorice	licochalcone a	HSP90AA1
Licorice	Inermine	HSP90AA1
Licorice	DFV	HSP90AA1
Licorice	Medicarpin	HSP90AA1
Licorice	Lupiwighteone	HSP90AA1
Licorice	7-Methoxy-2-methyl isoflavone	HSP90AA1
Licorice	naringenin	HSP90AA1
Licorice	glyasperin B	HSP90AA1
Licorice	glyasperin F	HSP90AA1
Licorice	Isotrifoliol	HSP90AA1
Licorice	Semilicoisoflavone B	HSP90AA1
Licorice	Glepidotin A	HSP90AA1
Licorice	Glepidotin B	HSP90AA1
Licorice	Glypallichalcone	HSP90AA1
Licorice	8-(6-hydroxy-2-benzofuranyl)-2,2-dimethyl-5-chromenol	HSP90AA1
Licorice	Licochalcone B	HSP90AA1
Licorice	licochalcone G	HSP90AA1
Licorice	Gancaonin A	HSP90AA1
Licorice	Gancaonin B	HSP90AA1
Licorice	3-(3,4-dihydroxyphenyl)-5,7-dihydroxy-8-(3-methylbut-2-enyl)chromone	HSP90AA1
Licorice	5,7-dihydroxy-3-(4-methoxyphenyl)-8-(3-methylbut-2-	HSP90AA1
Licorice	2-(3,4-dihydroxyphenyl)-5,7-dihydroxy-6-(3-methylbut-2-enyl)chromone	HSP90AA1
Licorice	Licocoumarone	HSP90AA1
Licorice	Licoisoflavone	HSP90AA1
Licorice	licoisoflavanone	HSP90AA1
Licorice	(E)-3-[3,4-dihydroxy-5-(3-methylbut-2-enyl)phenyl]-1-(2,4-dihydroxyphenyl)prop-2-en-1-one	HSP90AA1
Licorice	Glyzaglabrin	HSP90AA1
Licorice	Glabranin	HSP90AA1
Licorice	1,3-dihydroxy-9-methoxy-6-benzofurano[3,2-c]chromenone	HSP90AA1
Licorice	1,3-dihydroxy-8,9-dimethoxy-6-benzofurano[3,2-c]chromenone	HSP90AA1
Licorice	Eurycarpin A	HSP90AA1
Licorice	Sigmoidin-B	HSP90AA1
Licorice	(2R)-7-hydroxy-2-(4-hydroxyphenyl)chroman-4-one	HSP90AA1
Licorice	(2S)-7-hydroxy-2-(4-hydroxyphenyl)-8-(3-methylbut-2-enyl)chroman-4-one	HSP90AA1
Licorice	Isolicoflavonol	HSP90AA1
Licorice	1-Methoxyphaseollidin	HSP90AA1
Licorice	Quercetin der.	HSP90AA1
Licorice	6-prenylated eriodictyol	HSP90AA1
Licorice	7-Acetoxy-2-methylisoflavone	HSP90AA1
Licorice	8-prenylated eriodictyol	HSP90AA1
Licorice	Gancaonin G	HSP90AA1
Licorice	Gancaonin H	HSP90AA1

Licorice	Licoagrocarpin	HSP90AA1
Licorice	Glyasperins M	HSP90AA1
Licorice	Odoratin	HSP90AA1
Licorice	Phaseol	HSP90AA1
Chimonanthus salicifolius	kaempferol	HSP90AA1
Microctis Folium	isorhamnetin	PTGS2
Microctis Folium	kaempferol	PTGS2
Microctis Folium	4',5-Dihydroxyflavone	PTGS2
Polygonum chinense	kaempferol	PTGS2
Polygonum chinense	kaempferol-7-O-glucoside	PTGS2
Polygonum chinense	isorhamnetin	PTGS2
Polygonum chinense	luteolin	PTGS2
Ecliptae Herba	acacetin	PTGS2
Ecliptae Herba	butin	PTGS2
Ecliptae Herba	3'-O-Methylorobol	PTGS2
Ecliptae Herba	Pratensein	PTGS2
Ecliptae Herba	demethylwedelolactone	PTGS2
Ecliptae Herba	luteolin	PTGS2
Perilla Frutescens	luteolin	PTGS2
Isatidis Radix	acacetin	PTGS2
Isatidis Radix	EUPATORIN	PTGS2
Isatidis Radix	Dinatin	PTGS2
Isatidis Radix	quindoline	PTGS2
Isatidis Radix	hydroxyindirubin	PTGS2
Isatidis Radix	Indigo	PTGS2
Isatidis Radix	(2Z)-2-(2-oxoindolin-3-ylidene)indolin-3-one	PTGS2
Isatidis Radix	2-(9-((3-methyl-2-oxopent-3-en-1-yl)oxy)-2-oxo-1,2,8,9-tetrahydrofuro[2,3-h]quinolin-8-yl)propan-2-yl acetate	PTGS2
Isatidis Radix	DFV	PTGS2
Isatidis Radix	(E)-2-[(3-indole)cyanomethylene-]-3-	PTGS2
Isatidis Radix	neohesperidin_qt	PTGS2
Isatidis Radix	Sinensetin	PTGS2
Isatidis Radix	6-(3-oxoindolin-2-ylidene)indolo[2,1-b]quinazolin-12-one	PTGS2
Isatidis Radix	(E)-3-(3,5-dimethoxy-4-hydroxy-benzylidene)-2-indolinone	PTGS2
Isatidis Radix	(E)-3-(3,5-dimethoxy-4-hydroxyb-enzylidene)-2-indolinone	PTGS2
Isatidis Radix	3-[(3,5-dimethoxy-4-oxo-1-cyclohexa-2,5-dienylidene)methyl]-2,4-dihydro-1H-pyrrolo[2,1-b]quinazolin-9-one	PTGS2
Chrysanthemi Flos	[(1S,5S,7S)-7-acetoxy-5-isopropenyl-2,8-dimethylene-cyclodecyl] acetate	PTGS2
Chrysanthemi Flos	acacetin	PTGS2
Chrysanthemi Flos	Chryseriol	PTGS2
Chrysanthemi Flos	isorhamnetin	PTGS2
Chrysanthemi Flos	kaempferol	PTGS2
Chrysanthemi Flos	5,7-dihydroxy-2-(3-hydroxy-4-methoxyphenyl)chroman-4-one	PTGS2
Chrysanthemi Flos	luteolin	PTGS2
Chrysanthemi Flos	EUPATORIN	PTGS2
Chrysanthemi Flos	Diosmetin	PTGS2
Chrysanthemi Flos	naringenin	PTGS2

Chrysanthemi Flos	Artemetin	PTGS2
Licorice	Jaranol	PTGS2
Licorice	isorhamnetin	PTGS2
Licorice	formononetin	PTGS2
Licorice	Calycosin	PTGS2
Licorice	kaempferol	PTGS2
Licorice	licochalcone a	PTGS2
Licorice	Inermine	PTGS2
Licorice	DFV	PTGS2
Licorice	Glycyrol	PTGS2
Licorice	Medicarpin	PTGS2
Licorice	Lupiwighteone	PTGS2
Licorice	7-Methoxy-2-methyl isoflavone	PTGS2
Licorice	naringenin	PTGS2
Licorice	glyasperin B	PTGS2
Licorice	glyasperin F	PTGS2
Licorice	Isotrifoliol	PTGS2
Licorice	(E)-1-(2,4-dihydroxyphenyl)-3-(2,2-dimethylchromen-6-yl)prop-2-en-1-one	PTGS2
Licorice	(2S)-6-(2,4-dihydroxyphenyl)-2-(2-hydroxypropan-2-yl)-4-methoxy-2,3-dihydrofuro[3,2-	PTGS2
Licorice	Semilicoisoflavone B	PTGS2
Licorice	Glepidotin A	PTGS2
Licorice	Glepidotin B	PTGS2
Licorice	Glypallichalcone	PTGS2
Licorice	8-(6-hydroxy-2-benzofuranyl)-2,2-dimethyl-5-chromenol	PTGS2
Licorice	Licochalcone B	PTGS2
Licorice	licochalcone G	PTGS2
Licorice	Licoricone	PTGS2
Licorice	Gancaonin A	PTGS2
Licorice	Gancaonin B	PTGS2
Licorice	3-(3,4-dihydroxyphenyl)-5,7-dihydroxy-8-(3-methylbut-2-enyl)chromone	PTGS2
Licorice	5,7-dihydroxy-3-(4-methoxyphenyl)-8-(3-methylbut-2-	PTGS2
Licorice	2-(3,4-dihydroxyphenyl)-5,7-dihydroxy-6-(3-methylbut-2-enyl)chromone	PTGS2
Licorice	Licoisoflavone	PTGS2
Licorice	Licoisoflavone B	PTGS2
Licorice	licoisoflavanone	PTGS2
Licorice	shinpterocarpin	PTGS2
Licorice	(E)-3-[3,4-dihydroxy-5-(3-methylbut-2-enyl)phenyl]-1-(2,4-dihydroxyphenyl)prop-2-en-1-one	PTGS2
Licorice	Glyzaglabrin	PTGS2
Licorice	Glabranin	PTGS2
Licorice	Glabrone	PTGS2
Licorice	Eurycarpin A	PTGS2
Licorice	Sigmoidin-B	PTGS2
Licorice	(2R)-7-hydroxy-2-(4-hydroxyphenyl)chroman-4-one	PTGS2
Licorice	(2S)-7-hydroxy-2-(4-hydroxyphenyl)-8-(3-methylbut-2-enyl)chroman-4-one	PTGS2
Licorice	Isoglycyrol	PTGS2

Licorice	Isolicoflavonol	PTGS2
Licorice	HMO	PTGS2
Licorice	1-Methoxyphaseollidin	PTGS2
Licorice	Quercetin der.	PTGS2
Licorice	6-prenylated eriodictyol	PTGS2
Licorice	7-Acetoxy-2-methylisoflavone	PTGS2
Licorice	8-prenylated eriodictyol	PTGS2
Licorice	Gancaonin G	PTGS2
Licorice	Gancaonin H	PTGS2
Licorice	Licoagrocarpin	PTGS2
Licorice	Glyasperins M	PTGS2
Licorice	Licoagroisoflavone	PTGS2
Licorice	Odoratin	PTGS2
Licorice	Phaseol	PTGS2
Licorice	Xambioona	PTGS2
Chimonanthus salicifolius	kaempferol	PTGS2
Polygonum chinense	luteolin	CASP9
Ecliptae Herba	luteolin	CASP9
Perilla Frutescens	luteolin	CASP9
Chrysanthemi Flos	luteolin	CASP9
Chrysanthemi Flos	naringenin	MAPK3
Licorice	naringenin	MAPK3
Microctis Folium	kaempferol	MAPK8
Polygonum chinense	kaempferol	MAPK8
Chrysanthemi Flos	kaempferol	MAPK8
Licorice	kaempferol	MAPK8
Chimonanthus salicifolius	kaempferol	MAPK8
Polygonum chinense	Quercetin	EGFR
Polygonum chinense	3-O-methylellagic acid	EGFR
Polygonum chinense	kaempferol-7-O-glucoside	EGFR
Polygonum chinense	luteolin	EGFR
Ecliptae Herba	luteolin	EGFR
Perilla Frutescens	luteolin	EGFR
Chrysanthemi Flos	luteolin	EGFR
Chimonanthus salicifolius	Quercetin	EGFR
Polygonum chinense	luteolin	IL6
Ecliptae Herba	luteolin	IL6
Perilla Frutescens	luteolin	IL6
Chrysanthemi Flos	luteolin	IL6
Polygonum chinense	kaempferol-7-O-glucoside	TNF
Chimonanthus salicifolius	luteolin-5-O-glucoside	TNF
Polygonum chinense	luteolin	NFKBIA
Ecliptae Herba	luteolin	NFKBIA
Perilla Frutescens	luteolin	NFKBIA
Chrysanthemi Flos	luteolin	NFKBIA
Polygonum chinense	luteolin	MCL1
Ecliptae Herba	luteolin	MCL1
Perilla Frutescens	luteolin	MCL1
Chrysanthemi Flos	luteolin	MCL1
Microctis Folium	isorhamnetin	RELA
Microctis Folium	kaempferol	RELA
Polygonum chinense	kaempferol	RELA
Polygonum chinense	isorhamnetin	RELA
Polygonum chinense	luteolin	RELA
Ecliptae Herba	acacetin	RELA
Ecliptae Herba	luteolin	RELA
Perilla Frutescens	luteolin	RELA
Isatidis Radix	acacetin	RELA

Chrysanthemi Flos	acacetin	RELA
Chrysanthemi Flos	isorhamnetin	RELA
Chrysanthemi Flos	kaempferol	RELA
Chrysanthemi Flos	luteolin	RELA
Chrysanthemi Flos	naringenin	RELA
Licorice	isorhamnetin	RELA
Licorice	kaempferol	RELA
Licorice	licochalcone a	RELA
Licorice	naringenin	RELA
Chimonanthus salicifolius	kaempferol	RELA
Microctis Folium	isorhamnetin	ESR1
Polygonum chinense	isorhamnetin	ESR1
Ecliptae Herba	3'-O-Methylorobol	ESR1
Ecliptae Herba	Pratensein	ESR1
Ecliptae Herba	wedelolactone	ESR1
Isatidis Radix	hydroxyindirubin	ESR1
Isatidis Radix	(2Z)-2-(2-oxoindolin-3-ylidene)indolin-3-one	ESR1
Isatidis Radix	DFV	ESR1
Isatidis Radix	(E)-2-[(3-indole)cyanomethylene-]-3-	ESR1
Isatidis Radix	6-(3-oxoindolin-2-ylidene)indolo[2,1-b]quinazolin-12-one	ESR1
Chrysanthemi Flos	Chryseriol	ESR1
Chrysanthemi Flos	isorhamnetin	ESR1
Chrysanthemi Flos	naringenin	ESR1
Chrysanthemi Flos	Artemetin	ESR1
Licorice	isorhamnetin	ESR1
Licorice	formononetin	ESR1
Licorice	Calycosin	ESR1
Licorice	licochalcone a	ESR1
Licorice	DFV	ESR1
Licorice	Glycyrol	ESR1
Licorice	Medicarpin	ESR1
Licorice	Lupiwighteone	ESR1
Licorice	7-Methoxy-2-methyl isoflavone	ESR1
Licorice	naringenin	ESR1
Licorice	glyasperin B	ESR1
Licorice	glyasperin F	ESR1
Licorice	Isotrifoliol	ESR1
Licorice	(E)-1-(2,4-dihydroxyphenyl)-3-(2,2-dimethylchromen-6-yl)prop-2-en-1-one	ESR1
Licorice	(2S)-6-(2,4-dihydroxyphenyl)-2-(2-hydroxypropan-2-yl)-4-methoxy-2,3-dihydrofuro[3,2-	ESR1
Licorice	Semilicoisoflavone B	ESR1
Licorice	Glepidotin A	ESR1
Licorice	Glepidotin B	ESR1
Licorice	Glypallichalcone	ESR1
Licorice	8-(6-hydroxy-2-benzofuranyl)-2,2-dimethyl-5-chromenol	ESR1
Licorice	Licochalcone B	ESR1
Licorice	licochalcone G	ESR1
Licorice	Licoricone	ESR1
Licorice	Gancaonin A	ESR1
Licorice	Gancaonin B	ESR1

Licorice	3-(3,4-dihydroxyphenyl)-5,7-dihydroxy-8-(3-methylbut-2-enyl)chromone	ESR1
Licorice	5,7-dihydroxy-3-(4-methoxyphenyl)-8-(3-methylbut-2-	ESR1
Licorice	Licocoumarone	ESR1
Licorice	Licoisoflavone	ESR1
Licorice	Licoisoflavone B	ESR1
Licorice	licoisoflavanone	ESR1
Licorice	shinpterocarpin	ESR1
Licorice	(E)-3-[3,4-dihydroxy-5-(3-methylbut-2-enyl)phenyl]-1-(2,4-dihydroxyphenyl)prop-2-en-1-one	ESR1
Licorice	Glyzaglabrin	ESR1
Licorice	Glabranin	ESR1
Licorice	Glabrone	ESR1
Licorice	1,3-dihydroxy-9-methoxy-6-benzofurano[3,2-c]chromenone	ESR1
Licorice	1,3-dihydroxy-8,9-dimethoxy-6-benzofurano[3,2-c]chromenone	ESR1
Licorice	Eurycarpin A	ESR1
Licorice	Sigmoidin-B	ESR1
Licorice	(2R)-7-hydroxy-2-(4-hydroxyphenyl)chroman-4-one	ESR1
Licorice	(2S)-7-hydroxy-2-(4-hydroxyphenyl)-8-(3-methylbut-2-enyl)chroman-4-one	ESR1
Licorice	Isoglycyrol	ESR1
Licorice	Isolicoflavonol	ESR1
Licorice	HMO	ESR1
Licorice	1-Methoxyphaseollidin	ESR1
Licorice	Quercetin der.	ESR1
Licorice	6-prenylated eriodictyol	ESR1
Licorice	7-Acetoxy-2-methylisoflavone	ESR1
Licorice	8-prenylated eriodictyol	ESR1
Licorice	Gancaonin G	ESR1
Licorice	Gancaonin H	ESR1
Licorice	Licoagrocarpin	ESR1
Licorice	Glyasperins M	ESR1
Licorice	Licoagroisoflavone	ESR1
Licorice	Odoratin	ESR1
Licorice	Phaseol	ESR1
Licorice	Xambioona	ESR1
Licorice	formononetin	SIRT1
Microctis Folium	kaempferol	STAT1
Polygonum chinense	kaempferol	STAT1
Chrysanthemi Flos	kaempferol	STAT1
Licorice	kaempferol	STAT1
Chimonanthus salicifolius	kaempferol	STAT1
Microctis Folium	isorhamnetin	MAPK14
Polygonum chinense	isorhamnetin	MAPK14
Ecliptae Herba	3'-O-Methylorobol	MAPK14
Ecliptae Herba	Pratensein	MAPK14
Isatidis Radix	hydroxyindirubin	MAPK14
Isatidis Radix	(2Z)-2-(2-oxoindolin-3-ylidene)indolin-3-one	MAPK14
Isatidis Radix	(E)-2-[(3-indole)cyanomethylene-]-3-	MAPK14
Chrysanthemi Flos	Chryseriol	MAPK14
Chrysanthemi Flos	isorhamnetin	MAPK14

Chrysanthemi Flos	Artemetin	MAPK14
Licorice	isorhamnetin	MAPK14
Licorice	formononetin	MAPK14
Licorice	Calycosin	MAPK14
Licorice	licochalcone a	MAPK14
Licorice	Glycyrol	MAPK14
Licorice	Lupiwighteone	MAPK14
Licorice	7-Methoxy-2-methyl isoflavone	MAPK14
Licorice	glyasperin F	MAPK14
Licorice	Isotrifoliol	MAPK14
Licorice	(E)-1-(2,4-dihydroxyphenyl)-3-(2,2-dimethylchromen-6-yl)prop-2-en-1-one	MAPK14
Licorice	(2S)-6-(2,4-dihydroxyphenyl)-2-(2-hydroxypropan-2-yl)-4-methoxy-2,3-dihydrofuro[3,2-	MAPK14
Licorice	Glepidotin A	MAPK14
Licorice	Glypallichalcone	MAPK14
Licorice	Licochalcone B	MAPK14
Licorice	licochalcone G	MAPK14
Licorice	3-(3,4-dihydroxyphenyl)-5,7-dihydroxy-8-(3-methylbut-2-enyl)chromone	MAPK14
Licorice	5,7-dihydroxy-3-(4-methoxyphenyl)-8-(3-methylbut-2-	MAPK14
Licorice	Licoisoflavone	MAPK14
Licorice	shinpterocarpin	MAPK14
Licorice	(E)-3-[3,4-dihydroxy-5-(3-methylbut-2-enyl)phenyl]-1-(2,4-dihydroxyphenyl)prop-2-en-1-one	MAPK14
Licorice	Glyzaglabrin	MAPK14
Licorice	Glabrone	MAPK14
Licorice	1,3-dihydroxy-9-methoxy-6-benzofurano[3,2-c]chromenone	MAPK14
Licorice	1,3-dihydroxy-8,9-dimethoxy-6-benzofurano[3,2-c]chromenone	MAPK14
Licorice	Eurycarpin A	MAPK14
Licorice	HMO	MAPK14
Licorice	1-Methoxyphaseollidin	MAPK14
Licorice	Quercetin der.	MAPK14
Licorice	7-Acetoxy-2-methylisoflavone	MAPK14
Licorice	Gancaonin G	MAPK14
Licorice	Licoagrocarpin	MAPK14
Licorice	Licoagroisoflavone	MAPK14
Licorice	Odoratin	MAPK14
Licorice	Phaseol	MAPK14
